# The interplay between membrane lipids and phospholipase A family members in grapevine resistance against *Plasmopara viticola*

**DOI:** 10.1038/s41598-018-32559-z

**Published:** 2018-09-28

**Authors:** Gonçalo Laureano, Joana Figueiredo, Ana Rita Cavaco, Bernardo Duarte, Isabel Caçador, Rui Malhó, Marta Sousa Silva, Ana Rita Matos, Andreia Figueiredo

**Affiliations:** 1 Biosystems & Integrative Sciences Institute (BioISI), Faculdade de Ciências, Universidade de Lisboa, Campo Grande, 1749-016 Lisboa Portugal; 2 Laboratório de FTICR e Espectrometria de Massa Estrutural, Faculdade de Ciências, Universidade de Lisboa, Campo Grande, 1749-016 Lisboa Portugal; 3 Centro de Química e Bioquímica, Faculdade de Ciências, Universidade de Lisboa, Campo Grande, 1749-016 Lisboa Portugal; 4 MARE - Marine and Environmental Sciences Centre, Faculdade de Ciências, Universidade de Lisboa, Campo Grande, 1749-016 Lisboa Portugal

**Keywords:** Fatty acids, Biotic

## Abstract

Grapevine downy mildew, caused by the biotrophic oomycete *Plasmopara viticola*, is one of the most important diseases in modern viticulture. The search for sustainable disease control measure is of extreme importance, thus becoming imperative to fully characterize the mechanisms leading to an incompatible interaction. We have previously shown that lipid signalling events play an important role in grapevine’s response to this pathogen, namely through changes in linolenic acid content, lipid peroxidation and jasmonic acid synthesis. Here, we have characterized the modulation of lipid metabolism in leaves from two *V. vinifera* cultivars (resistant and susceptible to *P. viticola*) in the first hours after pathogen inoculation. Prior to pathogen inoculation both genotypes present an inherently different fatty acid composition that is highly modulated in the resistant genotype after pathogen challenge. Such changes involve modulation of phospholipase A activity suggesting that the source of lipids mobilized upon pathogen infection are the chloroplast membranes. This work thus provides original evidence on the involvement of lipid signalling and phospholipases in grapevine immune responses to pathogen infection. The results are discussed considering the implications on the plant’s physiological status and the use of discriminating lipid/fatty acids pattern in future selection procedures of cultivars.

## Introduction

Plant defence responses are activated by pathogen recognition and triggering of signalling cascades. In resistant plants, a rapid accumulation of reactive oxygen species (ROS), alteration of ion fluxes and production of defence compounds often lead to the establishment of a form of programmed cell death called hypersensitive reaction, restricting pathogen growth at the infection site^1^. During the establishment of these immune responses, plant hormones and oxylipins act as signals to trigger and mediate defence responses^2^. In recent years a growing number of evidences indicates that phospholipases and lipid associated molecules, namely glycerolipids, fatty acids, oxilipins and jasmonates, play essential roles in plant resistance (reviewed in^3^). Plant phospholipases, namely phospholipases A (PLA), catalyse the hydrolysis of phospholipids and galactolipids (the main component of plant membranes) into lysophospholipids and fatty acids^4^. The free fatty acids may then be oxidized by lypoxigenases leading to the biosynthesis of oxylipins and jasmonic acid (JA)^5^.

In our previous works we have shown strong evidences that grapevine resistance to the biotrophic oomycete *Plasmopara viticola*, the downy mildew causing agent, could be mediated on the first hours of interaction by JA and lipid associated signalling^6–8^. Indeed, we have shown that the content in the α-linolenic (C18:3) fatty acid is higher in resistant grapevine genotypes, further increasing after *P. viticola* inoculation^9^. The expression of key genes of JA synthesis is positively modulated after pathogen inoculation^6^ and both JA and JA-Ile content increase at 6 and 12 hours post-inoculation (hpi)^7^. Accumulation of proteins, such as the major latex protein-like protein 423 (MLP423), recently reported as associated to transmembrane lipid transport^10^ and non-specific lipid transfer proteins, associated to long distance signalling in systemic acquired resistance (SAR)^11,12^, suggest also an important role of lipids in grapevine resistance to *P. viticola*^8^.

Although several mechanisms associated to JA and lipid signalling in the grapevine-*P. viticola* pathosystem have been characterized, little is known on the first events after pathogen recognition leading to membrane lipid hydrolysis, increment of free C18:3 for JA biosynthesis and establishment of lipid associated signalling. We have conducted a characterization of fatty acid modulation in two grapevine genotypes (*V. vinifera* cv. Trincadeira and Regent, susceptible and resistant to *P. viticola*, respectively) in the first hours of interaction (6, 12 and 24 hpi). Moreover, we intended to investigate if PLA are also important in the establishment on the incompatible interaction between grapevine and *P. viticola*, by performing gene expression analysis. The selection of the PLA candidates required a thorough characterization of its superfamily in *V. vinifera*, inexistent so far. Thus, we have performed a genome wide analysis of this superfamily leading to the identification of grapevine PLA genes homologous to the previously described as associated to plant immunity in other plant models, but also located near the “*Resistance to Plasmopara viticola* (*Rpv*) loci”. Our work provides the first insights on the interplay between membrane lipids and PLA for the establishment of the incompatible interaction between grapevine and *P. viticola*.

## Results and Discussion

### Analysis of total fatty acids composition in grapevine-*P. viticola* interaction

Previously we have shown that a grapevine incompatible interaction with the biotrophic oomycete *Plasmopara viticola*, leads to JA biosynthesis, JA-Ile and H_2_O_2_ accumulation, and lipid peroxidation^6–8^. Although JA and lipid associated signalling events seem to play a crucial role in grapevine immunity, no characterization of *P. viticola*-induced FA modulation and lipid hydrolysis had been performed so far. Here we have analysed the FA composition of two grapevine genotypes (Regent and Trincadeira, resistant and susceptible to *P. viticola*, respectively) prior to pathogen challenge at the first hours of interaction.

**Figure 1:**
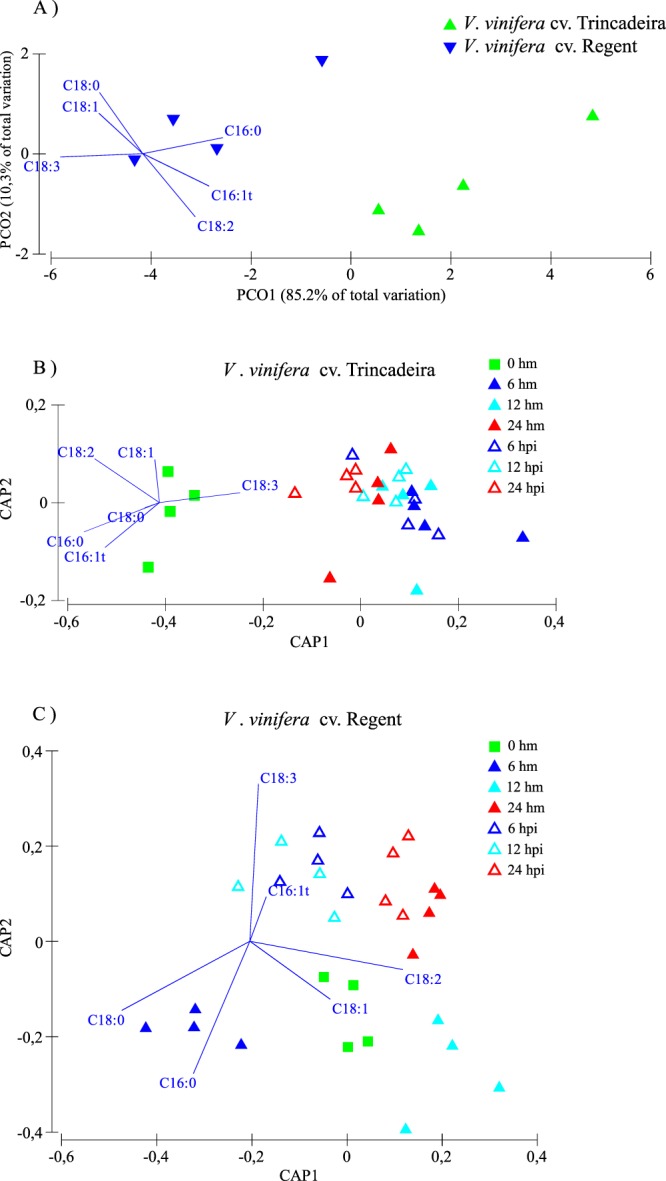
Canonical Analysis of Principal (CAP) coordinates plot based in the Euclidean distances between samples considering the complete leaves fatty acid profile of the mock inoculated groups of *V. vinifera* cv. Trincadeira and Regent (**A**), mock inoculated (hm) and inoculated (hpi) samples of Trincadeira (**B**) and Regent (**C**) varieties along the time course.

**Figure 2:**
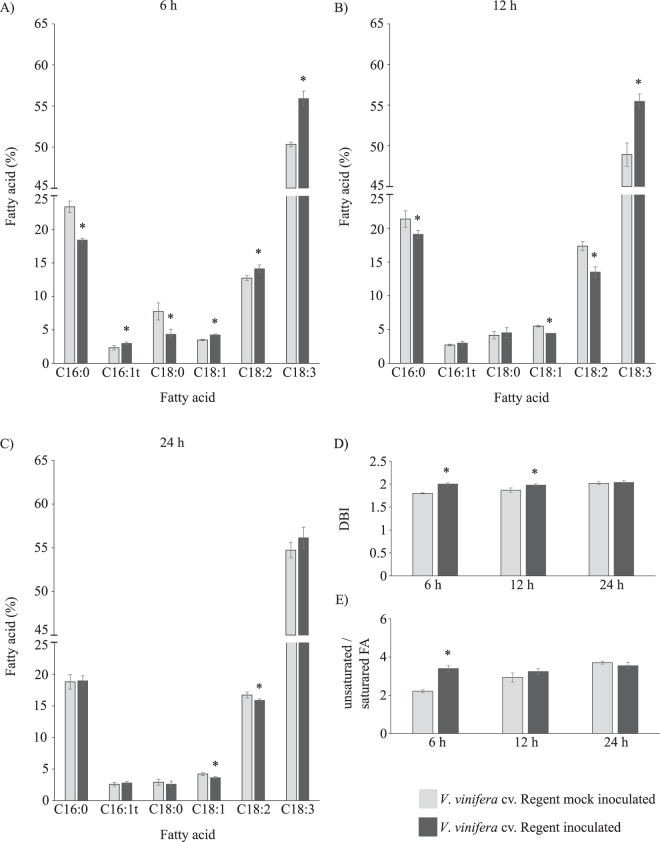
Fatty acid composition of *V*. *vinifera* cv. Regent mock inoculated (light grey) and inoculated (dark grey) leaves with *P. viticola* at 6 (**A**), 12 (**B**) and 24 (**C**) hours; (**D**) Double bound index (DBI); (**E**) Ratio between unsaturated and saturated FA. Values correspond to average relative percentage ± standard error, n = 4; Asterisks indicate significant differences (p < 0.05).

Before inoculation, both genotypes present a different FA content (Fig. 1A; Supplementary Fig. S1), with Regent presenting a higher accumulation of C18:3. After pathogen challenge, no significant alteration of FA content occurred in the susceptible genotype (Fig. 1B; Supplementary Fig. S2). However, several FA classes were significantly altered in the resistant genotype, particularly at 6 and 12 hpi (Figs 1C and 2). As shown in the Canonical Analysis of Principal (CAP) analysis, after inoculation the susceptible genotype does not follow a clear pattern of fatty acid modulation. Instead we observed a relatively constant pattern along time, leading to an overlap of all samples in a unique cloud (Fig. 1B). By contrast, in the resistant genotype, there is a clear evolution of the fatty acid remodelling after inoculation, with changes occurring mostly at 6 hpi. At 24 hpi fatty acid composition seems to evolve towards the basal composition becoming similar to mock inoculated samples. This can be interpreted as a clear sign of adaptation to the infection, consequence of a rearrangement of the lipid profile back to its homeostatic condition. At 6 hpi, both palmitic acid (C16:0) and stearic acid (C18:0) relative content decreased when compared to mock-inoculated samples, while the relative content of the unsaturated FA, oleic acid (C18:1), linoleic acid (C18:2) and C18:3 increased (Fig. 2A). Oleic acid is synthesized from C16:0 and a progressive desaturation of C18:0 leading to the formation of C18:1, C18:2 and C18:3 occurs^13^. Oleic acid was previously described to participate in plant defence mechanisms by stimulation or binding to proteins with an anti-cell-death effect^14^, increase of endogenous nitric oxide^15^ or azelaic acid (AzA) biosynthesis contributing to SAR^16,17^.

At 12 and 24 hpi both C18:1 and C18:2 relative content decreased while C18:3 relative content increased when compared to control samples (Fig. 2B,C). These results suggest that a desaturation of C18:0 leading to the accumulation of C18:3 is promoted after pathogen challenge. This is in agreement with Ali and co-workers, that detected a C18:3 accumulation in Regent inoculated with *P. viticola*^9^. High levels of unsaturated lipids had previously been associated to resistance against fungal and bacterial pathogens^5,13,18–20^ thus pointing out to the importance of 18 carbons’ FA in plant resistance. In fact, it is well known that in leaves the C18:3 fatty acid is mostly associated with the galactolipids, monogalactosyldiacylglycerol (MGDG) and digalactosyldiacylglycerol (DGDG), which account for more than 85% of thylakoid lipids^21^. The increase of C18:3 content in the first hours after *P. viticola* attack either associated to MGDG and DGDG may be associated to photosynthetic membranes protection, or lipid hydrolysis to obtain a free C18:3 fatty acid content for JA synthesis.

Modifications in the leaf FA content had a direct effect on the number of double-bounds present in the molecular structure of the fatty acids (Fig. 2D). The increase of unsaturation degree influences membrane fluidity and consequently membrane permeability^22^. Prior to pathogen challenge, when comparing the content in saturated and unsaturated lipids in the two grapevine genotypes, we observed that the unsaturated/saturated FA ratio is higher in Trincadeira than in Regent, together with a high double bound index (DBI) (Supplementary Fig. S1D,E). A higher DBI reflects an increase in membrane fluidity, thus our results suggest that innately the resistant genotype presents a more rigid membrane, when compared to the susceptible genotype, which may physically hinder pathogen entrance.

After inoculation, no alterations occur in Trincadeira. In Regent, both DBI and unsaturated/saturated ratios increase at 6 and 12 hpi (Fig. 2D,E; Supplementary Fig. S2D,E). The ability to adjust membrane lipid fluidity by changing levels of unsaturated fatty acids is a feature of stress acclimating plants maintaining a suitable environment for the function of integral proteins, such as the photosynthetic machinery^23^. Thus, we suggest that after pathogen challenge, the increase on membrane fluidity on the resistant genotype may be crucial to avoid membrane damage that represents inevitable effects on the energy transduction pathways and primary productivity^24^. Indeed, we have shown that a ROS burst occurs in Regent at 6 hpi^8^. Increase of DBI at the same time point may suggest that damage in membrane lipids is avoid by preventing ROS interaction with double bonds of the fatty acids. Moreover, we have shown that at 6 and 12 hpi there is a high turnover of photosynthetic proteins such as the ribulose bisphosphate carboxylase/oxygenase activase 1 (RCA1), ribulose-1-5-bisphosphate carboxylase (RuBisCO) small subunit and the photosystem II (PSII) stability/assembly factor HCF136 in Regent when compared to Trincadeira^8^. This may suggest that Regent is evoking defence mechanisms to protect and recover photosynthetic efficiency, upon *P. viticola* inoculation.

Recently it has also been suggested that a plant cuticle’s increased permeability may allow a quicker entrance of potential pathogen elicitors into the cells, triggering an accelerated and comprehensive defence response^25^. After cuticle, membranes are the next barrier to pathogen entrance, thus an increase of membrane permeability may also allow a faster perception of the pathogen.

### Lipid modulation during first hours of grapevine-*P. viticola* interaction

The major alterations on fatty acid profile occurred at 6 hpi after *P. viticola* infection, thus we have further evaluated the changes in lipid classes in both grapevine genotypes at this time-point. In accordance to the FA profile, prior to pathogen inoculation, both grapevine genotypes are innately different on leaf lipid composition (Supplementary Fig. S3). The resistant genotype, Regent, presents lower content of both MGDG and DGDG and higher content on all other lipid classes (phosphatidylglycerol (PG), phosphatidylcholine (PC), phosphatidylethanolamine (PE), phosphatidylglycerol (PI), phosphatidic acid (PA), FA, triacylglycerol (TAG) and other lipids) when compared to the susceptible genotype, Trincadeira (Supplementary Fig. S3).

**Figure 3:**
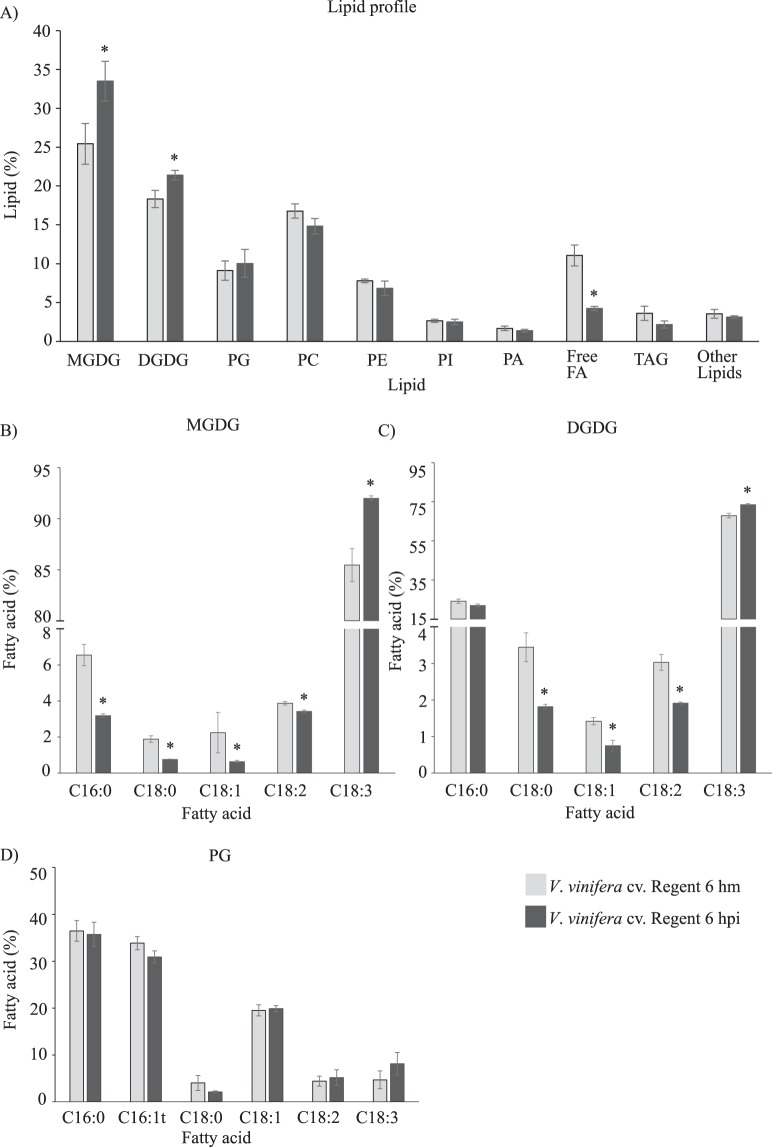
Lipid composition of *V*. *vinifera* cv. Regent mock inoculated (hm) and inoculated (hpi) leaves with *P. viticola* at 6 hours. (**A**) Total of lipids content; (**B**) Percentage of total FA present in MGDG; (**C**) Percentage of total FA in DGDG; (**D**) Percentage of total FA in PG. Values correspond to average relative percentage ± standard error, n = 3; Asterisks indicate significant differences (p < 0.05). Abbreviations: monogalactosyldiacylglycerol (MGDG), digalactosyldiacylglycerol (DGDG), phosphatidylglycerol (PG), phosphatidylcholine (PC), phosphatidylethanolamine (PE), phosphatidylglycerol (PI), phosphatidic acid (PA), free fatty acids (FA) and triacylglycerol (TAG).

After *P. viticola* inoculation, both genotypes behave differently. In Trincadeira, the distribution of lipid classes and their fatty acid composition were not significantly altered (Supplementary Fig. S4 and Table S1), while in Regent there is a significant modulation of both MGDG, DGDG, free FA and their FA composition (Fig. 3; Supplementary Table S2). The most significant changes concerned the two galactolipids, MGDG and DGDG. Their percentage increased from 43.8% in control plants to 54.9% in inoculated plants, indicating that lipid biosynthetic activities are occurring. The lipid content in C18:0, C18:1 and C18:2 decreased in MGDG and DGDG, while the content in C16:0 decreased only in MGDG (Fig. 3B,C). This FA modulation demonstrates that a desaturation of C18:0 may be occurring, leading to the accumulation of C18:3 in both galactolipids (Fig. 3B,C).

MGDG and DGDG are the main structural components of the thylakoid membranes^26^, although DGDG has also been recognized as important for the proper structure and function of PSII^27^. The proportions of PC and PG, the main phospholipid classes of leaf cell membranes, remain unchanged at 6 hpi (Fig. 3E; Supplementary Table S2). PG is the major phospholipid found in thylakoid membranes and it is likely to mediate interactions with the photosynthetic apparatus^28^, thus suggesting that photosynthesis remained impaired in Regent after pathogen challenge.

In both MGDG and DGDG, the content of C18:3 increased at 6 hpi in the inoculated samples (Fig. 3B,C). In fact, it is well known that in leaves, C18:3 is mostly associated with MGDG and DGDG, accounting for more than 85% of thylakoid lipids, fundamental for the photosynthetic metabolism^26^. The increase in the degree of unsaturated FA may also help to counteract the oxidative burst occurring in the resistant genotype after *P*. *viticola* inoculation^8^.

Free FA are originated from membrane glycerophospholipids through a hydrolysis process catalysed by phospholipases. Free FA may act as second messengers or as precursors of various oxylipins such as JA, with free C18:3 as substrate^29,30^. Also, free FA trigger a wide range of cellular responses, such as modulation of H^+^-ATPase in plasma membrane, that leads to cell wall acidification and activation of Mitogen Activated Protein Kinases (MAPK)^29^ or oxidative burst triggering^31^.

Constitutively, the resistant grapevine genotype presents a higher free FA content (Supplementary Fig. S3). Upon pathogen challenge, the content of free FA decreased in Regent, while in Trincadeira no significant alterations were observed (Fig. 3A, Supplementary Fig. S4). In the resistant genotype, the presence of higher free FA content by itself could be a significant factor to trigger a faster defence response at the moment of the interaction with pathogen. The decrease in free FA content after inoculation may suggest that these FA are involved in plant defence mechanisms, mainly as substrates for signalling molecules such as JA.

### Characterization of grapevine phospholipase A gene superfamily

In plants, the PLA superfamily comprise a group of enzymes with an important involvement in lipid signalling pathways through their ability to catalyse lipid hydrolysis leading to the release of fatty acids^4^. Plant PLA comprehend three major classes Phospholipase A_1_ (PLA_1_), comprising the defective in anther dehiscence (DAD) and PA-preferring PLA_1_, Secretory Phospholipase A_2_ (sPLA_2_) and Patatin-like Phospholipase A (pPLA)^29^.

The PLA superfamily has been already characterized in plants, namely on model species like *Arabidopsis thaliana* and rice^32,33^. In grapevine, we characterized this superfamily for the first time, using both Arabidopsis and rice PLA proteins as query for a BLAST against the *Vitis vinifera* genome. We also used the PLA conserved motifs as query to identify the PLA genes in grapevine genome, namely the catalytic centre GxSxG present in PLA_1_^4,34^, the sPLA_2_ Ca^2+^ binding loop YGKYCGxxxxGC, the active site DACCxxHDxC^35,36^, the pPLA anion-binding motif DGGGxRG and the esterase box GxSxG^32^.

**Figure 4:**
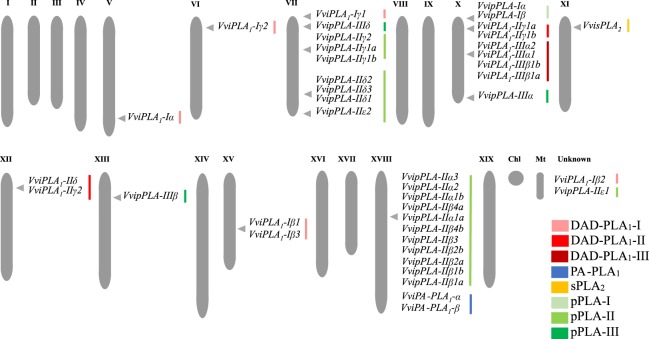
Locations of *Vitis vinifera* PLA genes in chromosomes. Proposed *V. vinifera* PLA nomenclature is shown in each chromosome.

A total of 41 PLA genes encoding for 43 predicted proteins were identified in *V. vinifera* (Supplementary Table S3). The number of gene members found in *V. vinifera* is higher than those reported for Arabidopsis, with 27 PLA genes, and rice, with 31 PLA genes^29,33^. All the identified grapevine PLA genes were mapped, being unevenly distributed in 9 of the 19 *V. vinifera* chromosomes (Fig. 4). The majority of PLA genes were located in chromosomes 7, 10 and 18. No PLA genes were detected in chromosomes 1, 2, 3, 4, 6, 8, 9, 14, 16, 17 and 19, and the specific location of 2 of the 41 grapevine PLA genes remains unknown (Fig. 4).

The exon-intron structure analysis of grapevine PLA genes reveals a pattern that allows discriminating members of each PLA group. The number of introns varies between 0 and 20, being 24% of the grapevine PLA genes intronless and around 15% presenting a high number of introns (12 to 20 introns), (Supplementary Table S3). All PLA genes without introns belong to the DAD-PLA_1_ family, which was also observed in Arabidopsis^4,33^. In Arabidopsis and rice the PLA members belonging to group I of DAD PLA_1_ are intronless^37,38^. In grapevine this intronless pattern is not observed in DAD PLA_1_ group I, due to the presence of one intron in *VviPLA1-Iγ1* (Supplementary Table S3), possibly due to an evolutionary process, but is found in group III (Supplementary Table S3). Both members of group I of the grapevine pPLA family have 18 introns, a value much higher than that observed in Arabidopsis^37,38^. On the other hand, the pPLA groups II and III, similarly to rice^33^, do not present a distinctive intron-exon pattern (Supplementary Table S3).

### Phylogenetic analysis of grapevine PLA

**Figure 5:**
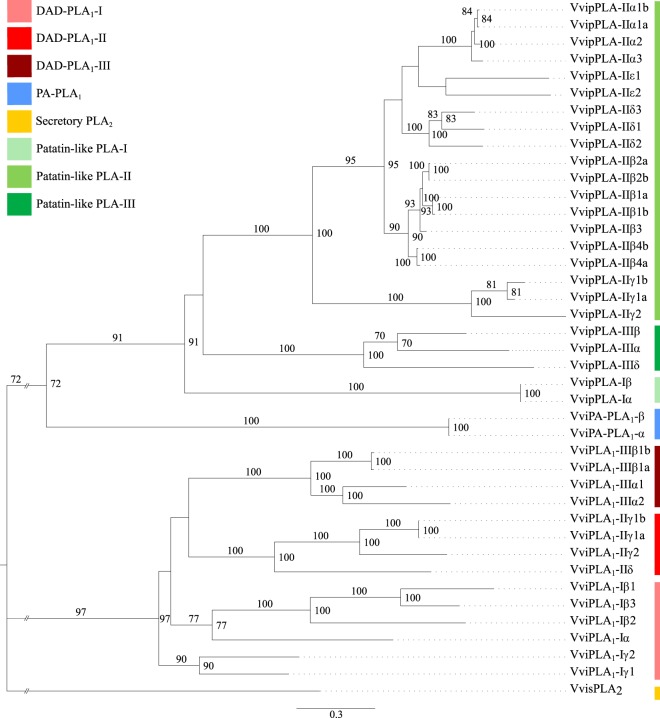
Maximum likelihood phylogenetic tree of the grapevine PLA superfamily. The numbers above branches show bootstrap values. Scale bar represents the number of estimated changes per branch length. Root was truncated with double dash totalling 0.3 changes per branch length.

In order to predict the structure of grapevine PLA superfamily, a phylogenetic analysis of both grapevine and Arabidopsis PLA proteins was performed (Supplementary Fig. S5–S7). With this analysis, we also propose a nomenclature for the members of the grapevine PLA superfamily (Fig. 4, indicated in each chromosome and Fig. 5) based on sequence identity with Arabidopsis PLA^29^ and the grapevine gene nomenclature method proposed by Grimplet and co-workers^39^.

The same phylogenetic analysis was also conducted considering only the 43 grapevine PLA proteins, to understand the phylogenetical relationship among them (Fig. 5). As expected, the grapevine PLA proteins are distributed in the three major classes, PLA_1_, sPLA_2_ and pPLA (Fig. 5; Supplementary Fig. S5–S7). The PLA_1_ class comprises DAD-PLA_1_ and PA-preferring PLA_1_ families. DAD-PLA_1_ class is divided into three groups, I, II and III, comprising 5, 4 and 4 members, respectively (Fig. 5; Supplementary Fig. S5). PA-preferring PLA_1_ class comprehends 2 members (Fig. 5; Supplementary Fig. S5). sPLA_2_ is the PLA family less represented with only one member (Fig. 5; Supplementary Fig. S6). On the other hand, pPLA is the most represented family, with 24 members, divided into three groups, I, II and III, with 2, 19 and 3 members, respectively (Fig. 5; Supplementary Fig. S7).

### Protein structure, domain analysis and subcellular targeting prediction

A prediction of the protein properties of grapevine PLA (Mw, pI, conserved motifs, domains and subcellular location) was performed.

Grapevine PLA proteins have a wide range of molecular weight, between 16 and 146 kDa (Supplementary Table S3), that varies accordingly to the Arabidopsis^4^ and rice^33^ PLA family. The DAD-PLA_1_ family proteins present a Mw range between 40 and 60 kDa, while PA-preferring PLA_1_ proteins members have a predicted Mw of 109 kDa (Supplementary Table S3). The pPLA family is characterized by a Mw between 40 and 50 kDa with exception for the pPLA group I with 2 proteins presenting a predicted Mw between 118 and 146 kDa (Supplementary Table S3). The PLA protein with the lowest predicted Mw is the secretory PLA with only 16 kDa (Supplementary Table S3).

Grapevine PLA theoretical pI varies between 5.02 and 9.51 (Supplementary Table S3), which is in accordance to the pI predicted for Arabidopsis PLA.

**Figure 6:**
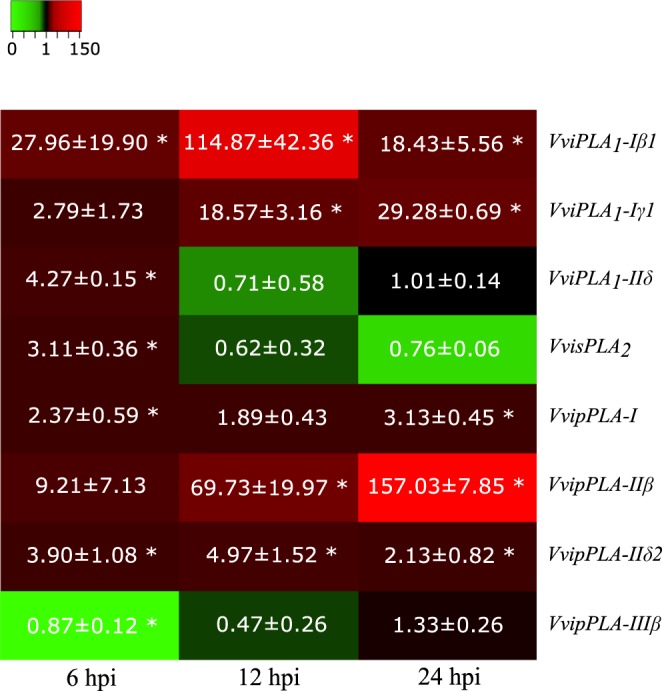
Multiple alignments of four grapevine PLA families representing the consensus and conserved motifs. Protein sequences were aligned for each PLA family, separately, applying MAFFT tool. The consensus motifs have been shown in shadow boxes according BLOSUM62. (**A**) VviPLA_1_; (**B**) VviPA-PLA_1_; (**C**) VvisPLA_2_; (**D**) VvipPLA.

Each PLA family has highly conserved motifs that are a distinctive feature. We have performed a multiple sequence alignment to identify the consensus and conserved motifs in each grapevine PLA family member (Fig. 6). As expected, all *V. vinifera* PLA_1_ share the highly conserved catalytic centre GxSxG^4,35,36^, (Fig. 6A,B). The sPLA_2_ family presented the highly conserved Ca^2+^ binding loop YGKYCGxxxxGC and the active site DACCxxHDxC, as previously described^4,29,34^, (Fig. 6C). All members of the patatin-like PLA presented the anion-binding motif DGGGxRG^32,33^, whereas the esterase box GxSxG^32,33^ was only found in pPLA groups I and II. In pPLA group III, the serine amino acid residue is replaced by a glycine presenting a non-canonic esterase box GxGxG, instead GxSxG. Specific motifs such as SAAPTY and DGGxxANN^33^ are present in all pPLA (Fig. 6D). The presence of these conserved motifs are in accordance to the previously described for rice PLA^33^.

Through the protein domain prediction analysis, we also found that all the grapevine DAD-PLA_1_ proteins present a lipase 3 domain (Supplementary Table S3) and that the PA-preferring PLA_1_ protein contain the DDHD domain, a conserved metal-binding site often seen in phosphoesterase domains (Supplementary Table S3). All pPLA contain the characteristic patatin domain, whilst the group I has an additional domain, the armadillo (ARM) repeats (Supplementary Table S3). Until now, no characteristic domain was identified for the sPLA_2_.

We have further analysed the predicted subcellular location for all the grapevine PLA proteins. The majority of the pPLA family members are located in cytosol, (Supplementary Table S3), as previously described^4^. Around 60% of the grapevine DAD-PLA_1_ are predicted to be located in the chloroplast and some members of the DAD-PLA1–II are also predicted to be located in the mitochondria (Supplementary Table S3). DAD-PLA groups I, II and III were previously described to be located in the chloroplast, cytosol and mitochondria, respectively^40^, however none of grapevine DAD-PLA were predicted to be located in the cytosol. The PA-preferring PLA_1_ are predicted to be located in the vacuole membrane (Supplementary Table S3).

### PLA gene expression correlates with free FA upon pathogen infection

In plants, the activity of PLA has been described in several stressful conditions associated to FA release from membranes by hydrolysis^4^. Free FA will act as substrates for the biosynthesis of signalling molecules, like JA^5^. Several PLA genes putatively involved in grapevine immunity were selected for expression analysis by qPCR, namely *VviPLA1-Iβ1, VviPLA1-Iγ1* and *VviPLA1-IIδ* (groups I and II of DAD-PLA_1_); *VvisPLA*_2_ (sPLA_2_) and *VvipPLA-I*, *VvipPLA-IIβ*, *VvipPLA-IIδ2* and *VvipPLA-IIIβ* (pPLA). PLA gene selection was based on sequence homology with Arabidopsis PLA genes involved in JA production, defence response and galactolipids’ metabolism, and their location close to several grapevine chromosomal loci associated with *P*. *viticola* resistance (named “*Resistance to P*. *viticola*” – *Rpv*, http://www.vivc.de/), (Supplementary Table S4).

Both FA and lipid profile suggested that the resistant grapevine genotype, Regent, presents the most significant changes after inoculation with *P. viticola*, suggesting that this cultivar may possess mechanisms to trigger an efficient defence response. Taking this into account, the gene expression profiles of the selected grapevine PLA genes were analysed in *V. vinifera* cv. Regent at 6, 12 and 24 hpi with *P. viticola*.

**Figure 7:**
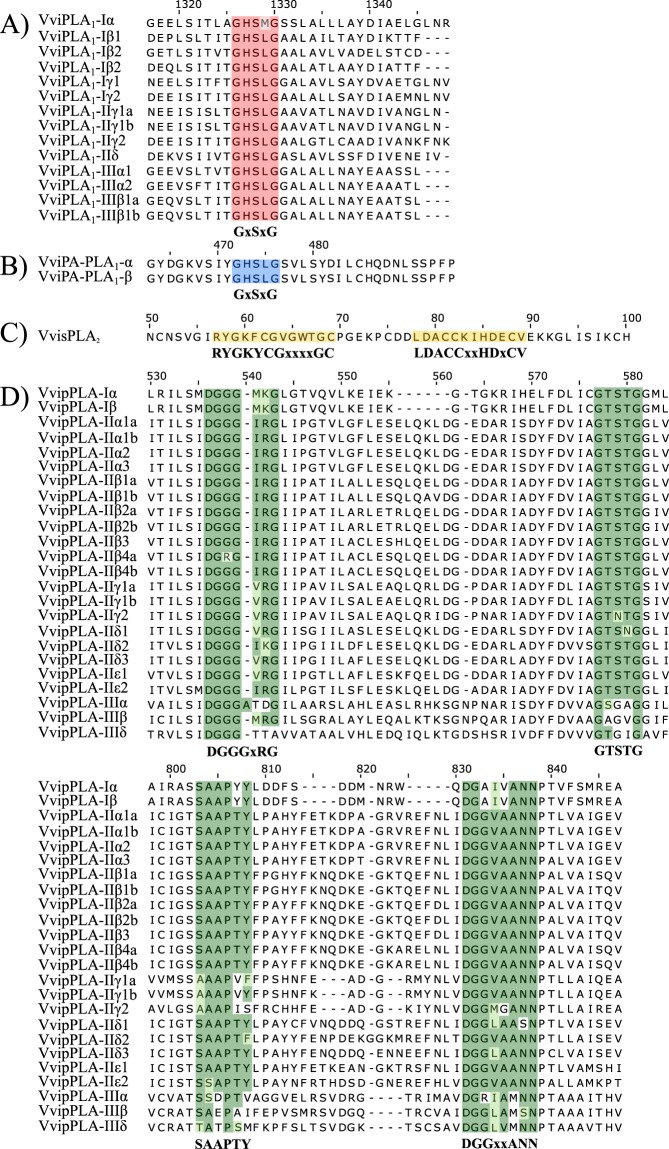
Gene expression profiles in Regent inoculated leaves. For each time point (6, 12 and 24 hpi) gene transcripts fold-change relative to controls are represented for *VviPLA*_*1*_-*Iβ1*; *VviPLA*_*1*_-*Iγ1*; *VviPLA*_*1*_-*IIδ*; *VvisPLA*_*2*_; *VvipPLA*-*I*; *VvipPLA*-*IIβ*; *VvipPLA*-*IIδ2*; *VvipPLA*-*IIIβ*. Fold-change values are relative to expression in mock inoculated leaves. Asterisks indicate significant differences (p < 0.05).

The selected grapevine PLA_1_ *VviPLA*1*-Iβ*1 and *VviPLA*1*-Iγ*1 present sequence homology with the Arabidopsis *AtPLA*_*1*_*Iβ1* and *AtPLA*_*1*_*Iγ*2, respectively, that encode chloroplastidial PLA_1_ involved in JA biosynthesis^41^. The expression of both *VviPLA1-Iβ1* and *VviPLA1-Iγ1* genes increased after *P. viticola* inoculation at all time-points, when compared to control leaves, with a higher expression of *VviPLA1-Iβ1* at 12 hpi (114.87 ± 42.36), (Fig. 7). In this cultivar, we previously reported an increase of JA levels after *P. viticola* infection, higher at 12 hpi^7^, consistent with the expression profile observed for *VviPLA1-Iβ1*, thus suggesting the involvement of this grapevine PLA_1_ gene in JA biosynthesis.

The *VviPLA*_*1*_*-IIδ* showed an up-regulation only at 6 hpi, decreasing its expression levels at 12 and 24 hpi. This gene shares sequence identity with Arabidopsis *AtPLA*_*1*_*IIδ*, which encodes for a chloroplastidial and cytoplasmatic PLA_1_ presenting a catabolic function and participating in leaf senescence^42^. Together with the fact that these genes encode proteins located in chloroplast and have hydrolytic activity, these PLA_1_ proteins may be responsible for FA release from galactolipids MGDG and DGDG, providing the biosynthetic precursors of both JA and AzA.

Within the patatin-like PLA family, we have selected genes from all the three groups, but only the members of groups I and II (*VvipPLA-I*, *VvipPLA-IIβ* and *VvipPLA-IIδ2*) presented an increased expression after pathogen challenge (Fig. 7). We highlight the gene expression modulation of *VvipPLA-IIβ* that highly increased its expression levels from 6 to 24 hpi (Fig. 7). Its Arabidopsis homologue *AtpPLA-II* is a cytosolic protein that catalyses the FA hydrolysis from phospholipids PG, PC and PI^43^. *VvipPLA-IIβ* is also predicted to be located in the cytosol (Supplementary Table S3) and may be linked to PC, PE, PI and PA hydrolysis, consistent with the decrease of these phospholipids’ levels immediately 6 hours after inoculation with *P. viticola* (Fig. 3). The patatin-like PLA genes *VvipPLA-I* and *VvipPLA-IIδ2* (located near to *Rpv9*) presented a slight expression increase at all time-points (Fig. 7). One of them, the *VvipPLA-I*, may be related to an enhanced protein activity culminating in FA release from galactolipids, considering that its Arabidopsis homologue was located both in cytosol and chloroplast^32^. The pPLA member from group III, *VvipPLA-IIIβ*, decreased its expression after *P. viticola* inoculation, returning to control levels at 24 hpi (Fig. 7). Its coding protein shares similarity with Arabidopsis AtpPLA-IIIβ, involved in phospholipids and galactolipids’ hydrolysis and possessing acyl-CoA thioesterase activity^44^. In Arabidopsis this protein increases its expression upon *Botritis cinerea* and *Pseudomonas syringae* inoculation, although its inactivation or overexpression did not alter pathogen resistance^45,46^. In the grapevine resistant genotype Regent, its homologue was repressed in response to *P. viticola*, so no clear relation could be established between this PLA and *P. viticola* resistance.

The expression of the secretory PLA *VvisPLA*_*2*_ increased immediately 6 hours after *P. viticola* infection, being repressed at the later time-points studied (Fig. 7). Its Arabidopsis homologue *AtsPLA*_2_ encodes for a small protein with increased activity in response to pathogen elicitor^47^ and associated to stomata opening under light condition^48^. sPLA_2_ proteins were described to act in several plant tissues and to trigger JA production, H^+^-ATPase stimulation, and stomata opening in stressful conditions^47,48^. Since *P. viticola* infects the grapevine leaf through the stomata^49^, the increased expression of *VvisPLA*_2_ at 6 hpi may be associated with the establishment of guard cell associated defence mechanisms in the early hours of interaction between grapevine and *P. viticola*, preventing the progression of infection.

## Conclusions

Our results provide new evidences for a lipid-associated signalling mechanism and lipid role in grapevine defence responses against *P. viticola*. The participation of several PLA in the resistance process and the existence of a discriminating lipid/FA pattern between resistant and susceptible grapevine genotypes were also highlighted.

By comparing both lipid and fatty acids profile in *V. vinifera* cv Regent and Trincadeira (resistant and susceptible to *P. viticola*, respectively), we were able to show a distinct constitutive profile as well as a differing lipid modulation in the first hours after inoculation with *P. viticola*. Despite the fact that constitutive FA profile may also reflect variability between the two genotypes under study, after pathogen challenge, only Regent presented a modulation of several lipid classes, suggesting that this genotype may trigger lipid-associated signalling mechanisms. C18:3, one of the JA precursors, is the most abundant FA in galactolipids and its levels increased in inoculated Regent leaves. Indeed, in the incompatible interaction Regent-*P. viticola*, JA plays an important role^6–8^, which, together with several lipid signalling events, will culminate in an effective defence response.

**Figure 8:**
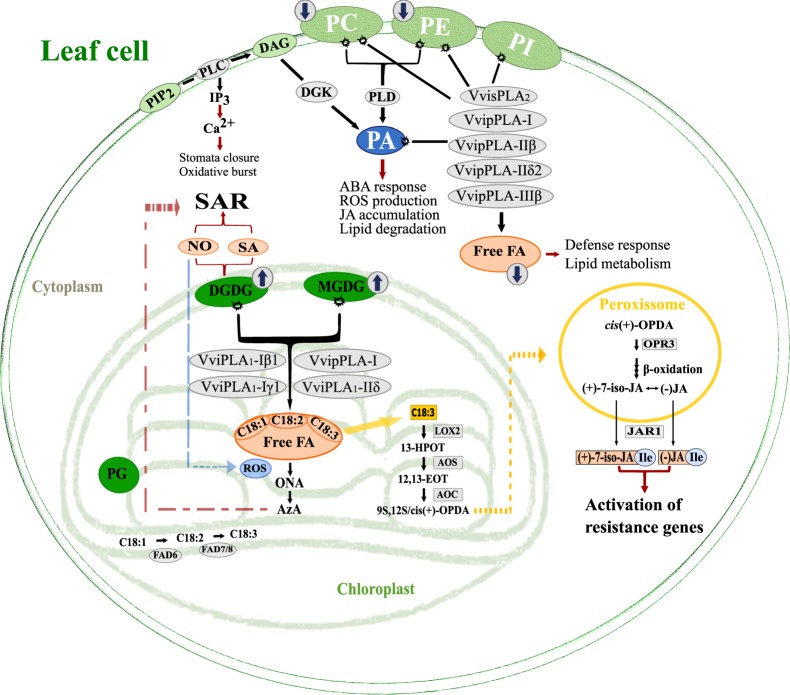
Lipid and FA modulation in *Vitis vinifera* cv. Regent at first hours upon infection with *P. viticola*. Fatty acids role in lipid signalling pathway, by phospholipases action, in plant defence mechanisms, upon its release from lipids, serving as signalling molecules or as substrate for oxylipins biosynthesis. Abbreviations: (9S,13S)-12-oxo-cis-10,15-phytodienoic acid (9S,13S/cis(+)-OPDA), 12,13-epoxy-9-Z,11,15-Z-octadecatrienoic acid (12,13-EOT), 13S-hydroperoxy-(9Z,11E,15)-octadecatrienoic acid (13-HPOT), (+)-7-iso-jasmonic acid ((+)-7-iso-JA), (−)-jasmonic acid ((−)-JA), abscisic acid (ABA), allene oxide cyclase (AOC), allene oxide synthase (AOS), azelaic acid (AzA), oleic acid (C18:1), linoleic acid (C18:2), α-linolenic acid (C18:3), calcium (Ca2^+^), diacylglycerol (DAG), di–galactosyldiacylglycerol (DGDG), diacylglycerol kinase (DGK), fatty acids desaturases 6/7/8 (FAD 6/7/8), inositol triphosphate (IP3), jasmonates-amide synthetase (JAR1), lipoxygenase 2 (LOX2), mono–galactosyldiacylglycerol (MGDG), nitric oxide (NO), phosphatidic acid (PA), phosphatidylcholine (PC), phosphatidylethanolamine (PE), phosphatidilglycerol (PG), phosphatidylinositol (PI), phosphatidylinositol 4,5-bisphosphate (PIP_2_), phospholipase C (PLC), phospholipase D (PLD), monocarboxylic acid 9-oxononanoic acid (ONA), oxophytodienoate reductase 3 (OPR3), reactive oxygen species (ROS), salicylic acid (SA), systemic acquired resistance (SAR).

Moreover, most of the PLA genes analysed showed an increased expression after pathogen challenge, particularly 6 hours after infection. Relevance should be given to the PLA_1_ gene *VviPLA1-Iβ1*, which expression profile is related to changes in JA levels, previously observed in Regent-*P. viticola* interaction^7^. This protein shares sequence identity with an Arabidopsis chloroplastidial PLA_1_ involved in JA biosynthesis^41^, suggesting that in grapevine it might also have the same function. Also, the secretory *VvisPLA*_2_, over-expressed at 6 hpi may be involved in regulation of stomata opening and JA biosynthesis^47,48^. Upon infection of *P. viticola* may trigger guard cell associated defence mechanisms at the early hours of interaction to prevent disease progression. Hence, PLA may have a direct role in grapevine resistance mechanisms against *P. viticola* by releasing FA from lipid membranes, generating free FA that will act directly as signalling molecules, or indirectly serving as biosynthetic precursors of AzA or JA (Fig. 8). On the other hand, secretory PLA may have a crucial role in the grapevine defence response that goes beyond lipid hydrolysis activity, also previously suggested for Arabidopsis and several animals (reviewed in^3^).

Altogether our results suggest an activation of lipid signalling in Regent and open new insights into the role of lipid molecules in plant-pathogen interactions. More studies must be conducted to fully characterize the involvement of lipids on this pathosystem.

## Materials and Methods

### Plant Material and Inoculation Experiments

Two *Vitis vinifera* cultivars with different resistant degrees to *P. viticola* were selected for this work. The *Vitis vinifera* cv Regent is a crossing line, bred for both downy and powdery mildew resistance at Julius Kuhn Institute (JKI, Germany), it presents the resistance to *P. viticola* loci 3.1 (RPV3.1) and presents a high degree of tolerance to both mildews^50^. *Vitis vinifera* cv Trincadeira is a traditional Portuguese grapevine cultivar widely used for quality wine production and highly sensitive to *P. viticola*^51^. Both cultivars were grown under identical greenhouse conditions, natural day/night rhythm with temperatures ranging between 5 and 28 °C, according to previously optimized conditions^52^. *P. viticola* sporangia were collected from symptomatic leaves from greenhouse infected plants after an overnight incubation in a moist chamber at room temperature, as previously described^52^. Sporangia were carefully collected by brushing, dried and stored at −25 °C. Their vitality was confirmed by microscopy^53^. A suspension containing 10^4^ sporangia.ml^−1^ was used to spray the abaxial leaf surface, while controls were made by spraying the leaves with water (mock inoculations). After inoculation, plants were kept for 8 h in a moist chamber (100% humidity) and then kept under greenhouse conditions during the inoculation time course. The third to fifth fully expanded leaves below the shoot apex were collected at 6, 12, and 24 hours post inoculation (hpi), and at the same time-points after mock inoculation (hm), immediately frozen in liquid nitrogen and stored at −80 °C. Five independent biological replicates were collected for each condition (inoculated and mock inoculated).

### Lipid Analysis

Ground leaves were boiled in water for 5 min to inactivate lipolytic enzymes. The extraction of lipophilic compounds was performed using a mixture of chloroform/methanol/water (1:1:1, v/v/v), as previously described^36^. Lipid classes’ separation was carried out at 6 hpi by thin layer chromatography (TLC) on silica plates (G-60, Merck, VWR) using a two- solvents’ system: chloroform/methanol/acetone/acetic acid/water (100/20/40/20/8, v/v/v/v/v) that separates the different polar lipids^54^, while the neutral lipids’ separation from polar lipids was carry out using the petroleum ether/ethyl ether/acetic acid (70/30/0.4, v/v/v) solvent mixture^55^. Lipids bands were visualized with 0.01% primuline in 80% acetone (v/v) under UV light, and scraped off. Fatty acids methyl esters (FAME) were prepared by trans-methylation of fatty acids with methanol:sulfuric acid (97.5:2.5, v/v) for 1 h at 70 °C. The methyl esters were recovered by adding petroleum ether:ultrapure water (3:2, v/v) and the organic phase was collected. Fatty acids quantitative analysis was performed using gas chromatography (430 Gas Chromatograph, Varian) at 210 °C, equipped with hydrogen flame ionization detector. Heptadecanoic acid (C17:0) was used as an internal standard. The double bond index (DBI) was calculated as follows:

$${\rm{DBI}}=( \% \,{\rm{monodienoic}}\,{\rm{acids}})+2\,( \% \,{\rm{dienoic}}\,{\rm{acids}})+3\,( \% \,{\rm{trienoic}}\,{\rm{acids}})/100.$$

### Identification and Retrieval of Grapevine PLA Sequences

Phospholipase A genes and putative protein sequences’ identification was performed using *Arabidopsis thaliana* and *Oryza sativa* (rice) PLA protein sequences as query for blast searches at NCBI BLAST tool (https://blast.ncbi.nlm.nih.gov/Blast.cgi)^56^. Arabidopsis and rice PLA members were searched and the sequences were retrieved from TAIR (https://arabidopsis.org)^57^ and RGAP (https://rice.plantbiology.msu.edu)^58^ databases, respectively. To find additional *Vitis vinifera* PLA, a search restricted to “*Vitis*” was performed on NCBI (https://www.ncbi.nlm.nih.gov/), using the PLA conserved motifs (GxSxG^4,34^, RYGKYCGxxxxGC, LDACCxxHDxCV^35,36^, DGGGxRG, GTSTG, SAAPTY, DGGxxANN^32,33^) as query (November 2017). The putative grapevine PLA sequences were further confirmed on CRIBI database (V2 annotation) (http://genomes.cribi.unipd.it/grape/)^59^.

### Domain Structure Analysis, Sequence Properties, Subcellular Location Prediction and Chromosomal Location

Domain and clan determination was performed using Pfam database (http://pfam.xfam.org/)^60^. Molecular weight (Mw) and isoelectric point (pI) were predicted using the ProtParam tool from ExPASy (http://web.expasy.org/protparam/)^61^. Subcellular location was predicted using TargetP (http://www.cbs.dtu.dk/services/TargetP/)^62^, Localizer (http://localizer.csiro.au/)^63^ and Predotar (https://urgi.versailles.inra.fr/predotar/)^64^ servers and PLA putative function using Blast2GO version 3.3 software tool (https://www.blast2go.com/)^65^. The Map Viewer tool from NCBI (http://www.ncbi.nlm.nih.gov/mapview/) was used to map PLA genes in *V. vinifera* chromosomes. The physical map constructed with grapevine phospholipase A genes’ location was also compared to a genetic linkage map representing *P. viticola* resistance (Resistance to *Plasmopara viticola*, *Rpv*) QTLs in grapevine^66–76^ to access the location of grapevine PLA within these loci. All the molecular predictions were manually curated and compiled.

### Phylogenetic Analysis

The MAFFT software, with the L-INS-I option version 7 (http://mafft.cbrc.jp/alignment/software/)^77^, was used for grapevine and Arabidopsis PLA protein sequences alignment. Sequences edition was performed with Jalview software (http://www.jalview.org/)^78^. A maximum likelihood (ML) phylogenetic analysis was obtained with RAxML-HPC v.8, on CIPRES Science Gateway (https://www.phylo.org)^79^, with the following parameters: protein substitution model PROTCAT; protein substitution model + BLOSUM62; bootstrap 1000 iterations with rapid boot strap analysis (−fa). Both trees were viewed on FIGTree (http://tree.bio.ed.ac.uk/software/figtree/) and edited on Inkscape (http://www.inkscape.org/).

### RNA Extraction and cDNA Synthesis

Total RNA was isolated from *V. vinifera* cv. Regent inoculated and mock inoculated frozen leaves using the Spectrum™ Plant Total RNA Kit (Sigma-Aldrich, USA). On-Column DNase I Digestion (Sigma-Aldrich, USA) was used to hydrolyse residual genomic DNA, as described by the manufacturer. RNA quality and concentration were determined using a NanoDrop-1000 spectrophotometer (Thermo Scientific), while integrity was analysed by agarose gel electrophoresis. Prior to complementary DNA (cDNA) synthesis, all samples were analysed for genomic DNA contamination by a quantitative real time Polymerase Chain Reaction (qPCR) of a reference gene on crude RNA^80^. Complementary DNA was synthesized from 2.5 µg of total RNA using RevertAid®H Minus Reverse Transcriptase (Fermentas, Ontario, Canada) anchored with Oligo(dT)23 primer (Fermentas, Ontario, Canada), following the manufacturer’s instructions.

### Quantitative Real Time PCR (qPCR)

qPCR experiments were performed in a StepOne™ Real-Time PCR system (Applied Biosystems, Sourceforge, USA) using the Maxima™ SYBR Green qPCR Master Mix (2×) kit (Fermentas, Ontario, Canada), following manufacturer’s instructions. Each reaction contained 2.5 mM MgCl_2_ and 2 µM of each primer were used in 25 µL volume reactions, with 4 µL of cDNA as template. A control without cDNA template was included in each set of reactions. Primer sequences are provided in Supplementary Table S5. For all genes, thermal cycling started with a 95 °C denaturation step for 10 minutes followed by 40 cycles of denaturation at 95 °C for 15 seconds and annealing at gene specific temperature (Supplementary Table S5) for 30 seconds. Dissociation curve analysis was performed to confirm single product amplification and the existence of non-specific PCR products (Supplementary Fig. S8). Three biological replicates and two technical replicates were used for each sample. Gene expression (fold change) was calculated as described in^81^. Elongation Factor 1-alpha (EF1α) and Ubiquitin-conjugating enzyme (UBQ) coding genes were used for expression data normalization as previously described^82^.

### Statistical analysis

Due to the lack of data normality and homogeneity of variances, the statistical analysis of the data was based on non-parametric tests. In order to compare FA, lipid profile and qPCR data in inoculated and mock inoculated samples, statistical analysis was performed by the Mann-Whitney U test using IBM^®^ SPSS^®^ Statistics software (version 23.0; SPSS Inc., USA). Results yielding p < 0.05 were considered statistically significant. In addition to the traditional univariate comparisons between fatty acid contents of each genotype, we also performed the evaluation of the whole lipid profile as one. Thus, the data was analysed using a multivariate approach that already proved to be an efficient tool to evaluate the fatty acid profile of plant ecotypes^24^. All statistical analysis were conducted using Primer 6 software^83^. Data regarding the total fatty acid relative composition of the tested cultivars were used to construct a resemblance matrix based in Euclidean distances. Canonical analysis of principal coordinates (CAP) was used to generate a statistical multivariate model based in fatty acid relative composition having this profile as modelling vectors, for each cultivar along the studied time points. As the CAP analysis is not efficient in describing populations composed by only two groups of samples, for the initial comparison between the two cultivars without pathogen exposure, a Principal Coordinate Analysis was undertaken instead^84^.

## Electronic supplementary material

Supplementary information
