# Author Correction: The interplay between membrane lipids and phospholipase A family members in grapevine resistance against *Plasmopara viticola*

**DOI:** 10.1038/s41598-019-42135-8

**Published:** 2019-04-25

**Authors:** Gonçalo Laureano, Joana Figueiredo, Ana Rita Cavaco, Bernardo Duarte, Isabel Caçador, Rui Malhó, Marta Sousa Silva, Ana Rita Matos, Andreia Figueiredo

**Affiliations:** 1 Biosystems & Integrative Sciences Institute (BioISI), Faculdade de Ciências, Universidade de Lisboa, Campo Grande, 1749-016 Lisboa Portugal; 2 Laboratório de FTICR e Espectrometria de Massa Estrutural, Faculdade de Ciências, Universidade de Lisboa, Campo Grande, 1749-016 Lisboa Portugal; 3 Centro de Química e Bioquímica, Faculdade de Ciências, Universidade de Lisboa, Campo Grande, 1749-016 Lisboa Portugal; 4 MARE - Marine and Environmental Sciences Centre, Faculdade de Ciências, Universidade de Lisboa, Campo Grande, 1749-016 Lisboa Portugal

Correction to: *Scientific Reports* 10.1038/s41598-018-32559-z, published online 28 September 2018

This Article contains an error in the order of the Figures. Figures 6 and 7 were published as Figures 7 and 6 respectively. The Figure legends are correct and the correct Figures 6 and 7 appear below as Figures 1 and 2.

**Figure 1:**
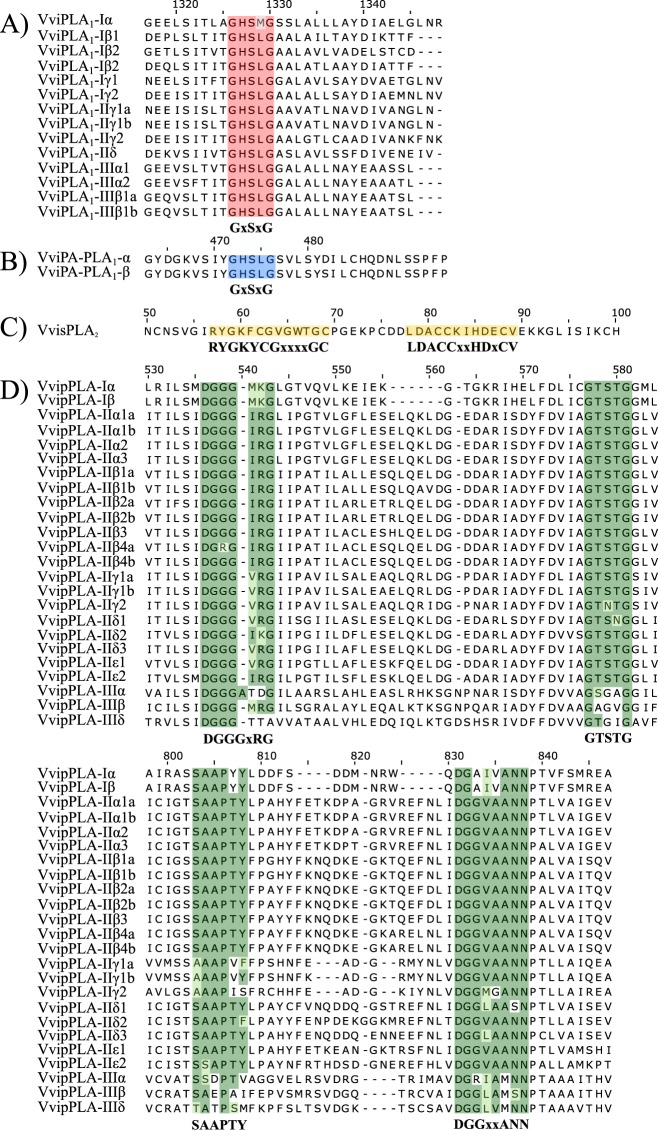
Multiple alignments of four grapevine PLA families representing the consensus and conserved motifs. Protein sequences were aligned for each PLA family, separately, applying MAFFT tool. The consensus motifs have been shown in shadow boxes according BLOSUM62. (**A**) VviPLA1; (**B**) VviPA-PLA1; (**C**) VvisPLA2; (**D**) VvipPLA.

**Figure 2:**
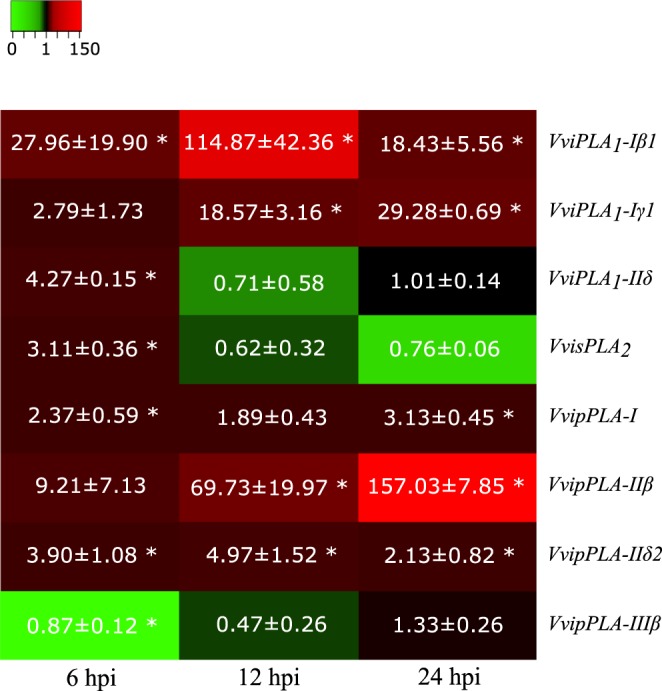
Gene expression profiles in Regent inoculated leaves. For each time point (6, 12 and 24 hpi) gene transcripts fold-change relative to controls are represented for *VviPLA*_*1*_*-Iβ1; VviPLA*_*1*_*-Iγ1; VviPLA*_*1*_*-IIδ;* *VvisPLA*_*2*_*; VvipPLA-I; VvipPLA-IIβ; VvipPLA-IIδ2; VvipPLA-IIIβ*. Fold-change values are relative to expression in mock inoculated leaves. Asterisks indicate significant differences (p < 0.05).

In addition, this Article contains a typographical error in the Results and Discussion section, under the subheading ‘Lipid modulation during first hours of grapevine-P. viticola interaction’ where,

“The proportions of PC and PG, the main phospholipid classes of leaf cell membranes, remain unchanged at 6 hpi (Fig. 3E; Supplementary Table S2)”.

should read:

“The proportions of PC and PG, the main phospholipid classes of leaf cell membranes, remain unchanged at 6 hpi (Fig. 3D; Supplementary Table S2)”.

