# Supplementary material for: The interplay between membrane lipids and phospholipase A family members in grapevine resistance against Plasmopara viticola
Source: Sci Rep. 2018 Sep 28;8:14538. doi: 10.1038/s41598-018-32559-z (PMC6162203; doi:10.1038/s41598-018-32559-z)
Supplement: Supplementary file 1 — Supplementary information [file 41598_2018_32559_MOESM1_ESM.pdf]

The interplay between membrane lipids and phospholipase A family members in grapevine resistance against *Plasmopara viticola*

Gonçalo Laureano [a], Joana Figueiredo [abc*], Ana Rita Cavaco [a*], Bernardo Duarte [d], Isabel Caçador [d], Rui Malhó [a], Marta Sousa Silva [bc], Ana Rita Matos [a†], Andreia Figueiredo [a†#]

[a] *Biosystems & Integrative Sciences Institute (BioISI), Faculdade de Ciências, Universidade de Lisboa, Campo Grande, 1749-016 Lisboa, Portugal*
[b] *Laboratório de FTICR e Espectrometria de Massa Estrutural, Faculdade de Ciências, Universidade de Lisboa, Campo Grande, 1749-016 Lisboa, Portugal*
[c] *Centro de Química e Bioquímica, Faculdade de Ciências, Universidade de Lisboa, Campo Grande, 1749-016 Lisboa, Portugal*
[d] *MARE - Marine and Environmental Sciences Centre, Faculdade de Ciências, Universidade de Lisboa, Campo Grande, 1749-016 Lisboa, Portugal*

* These authors contributed equally in this paper
† These authors are co-senior authors in this paper

Corresponding author:

[#]Andreia Figueiredo, at Biosystems & Integrative Sciences Institute (BioISI), Faculdade de Ciências da Universidade de Lisboa, 1749-016 Lisboa, Portugal. e-mail address: aafigueiredo@fc.ul.pt

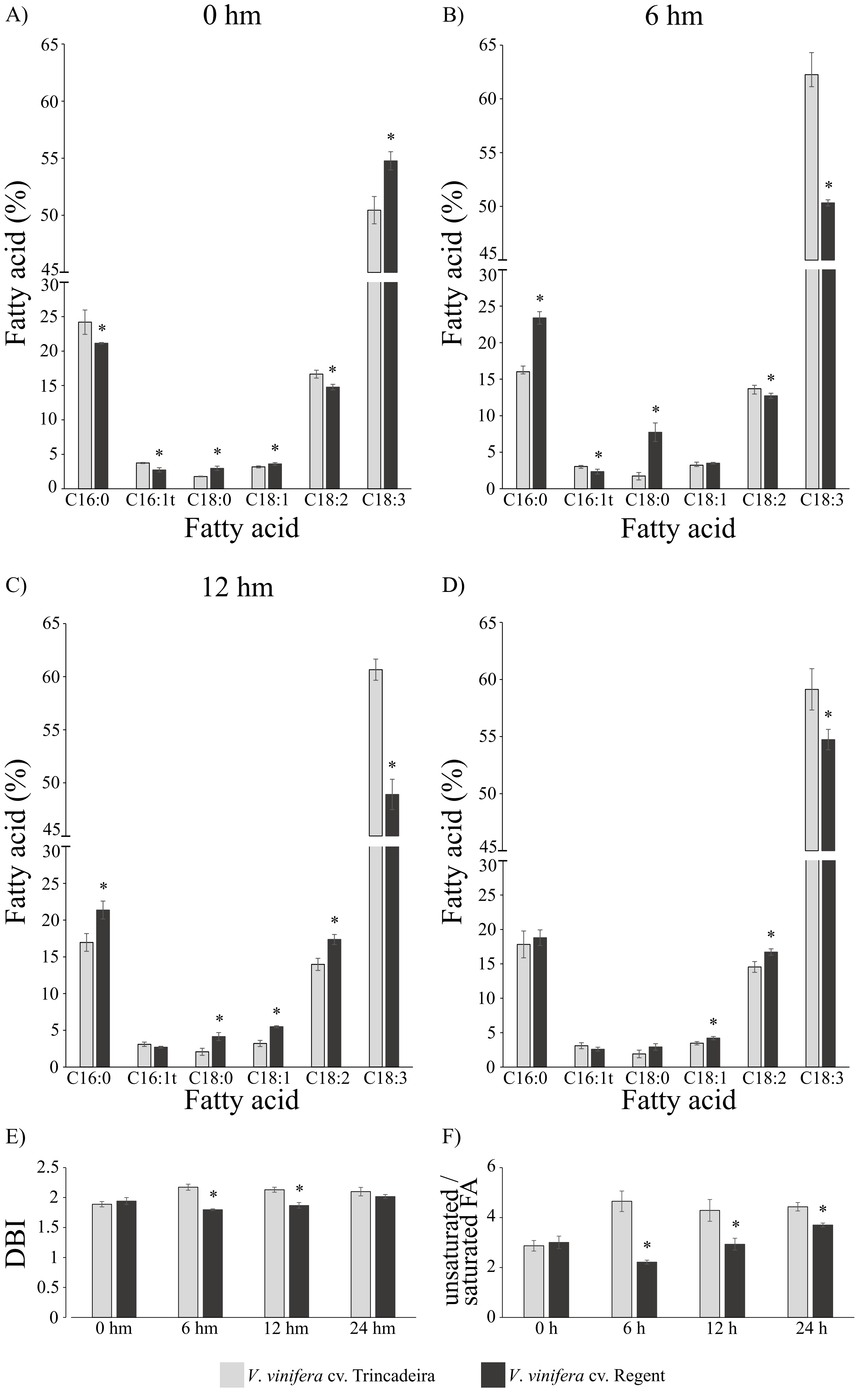

Supplementary Figure S1- Fatty acid composition between *V. vinifera* cv. Trincadeira (light grey) and *V. vinifera* cv. Regent (dark grey) mock inoculated leaves at 0 (A), 6 (B), 12 (C) and 24 (D) hours; E) Double bound index (DBI); F) Ratio between unsaturated and saturated FA. Values correspond to average relative percentage ± standard error, n = 4; Asterisks indicate significant differences ($p<0.05$).

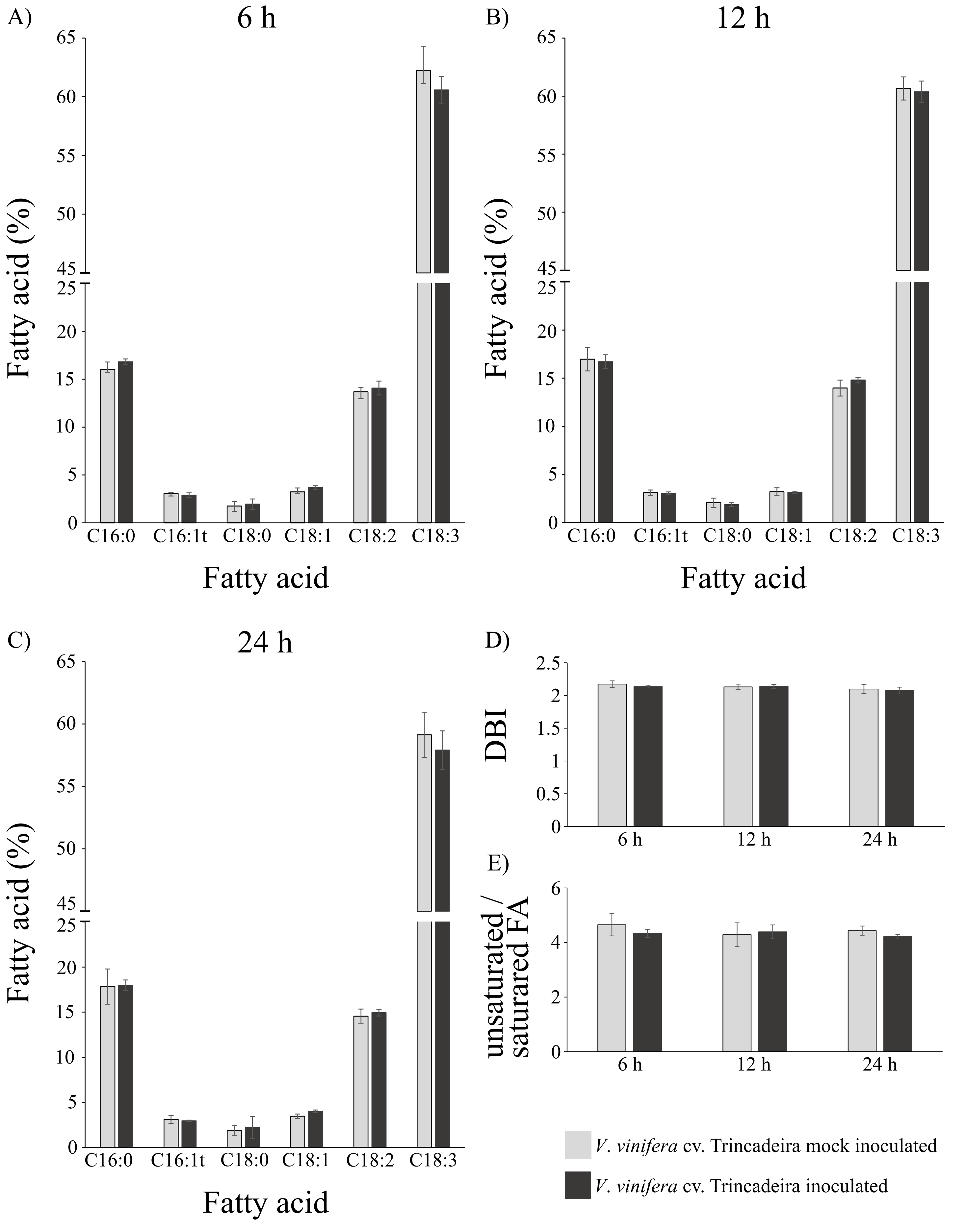

Supplementary Figure S2 - Fatty acid composition of *V. vinifera* cv. Trincadeira mock inoculated (light grey) and inoculated (dark grey) leaves with *P. viticola* at 6 (A), 12 (B) and 24 (C) hours; D) Double bound index (DBI); E) Ratio between unsaturated and saturated FA. Values correspond to average relative percentage ± standard error, n = 4; Asterisks indicate significant differences ($p<0.05$).

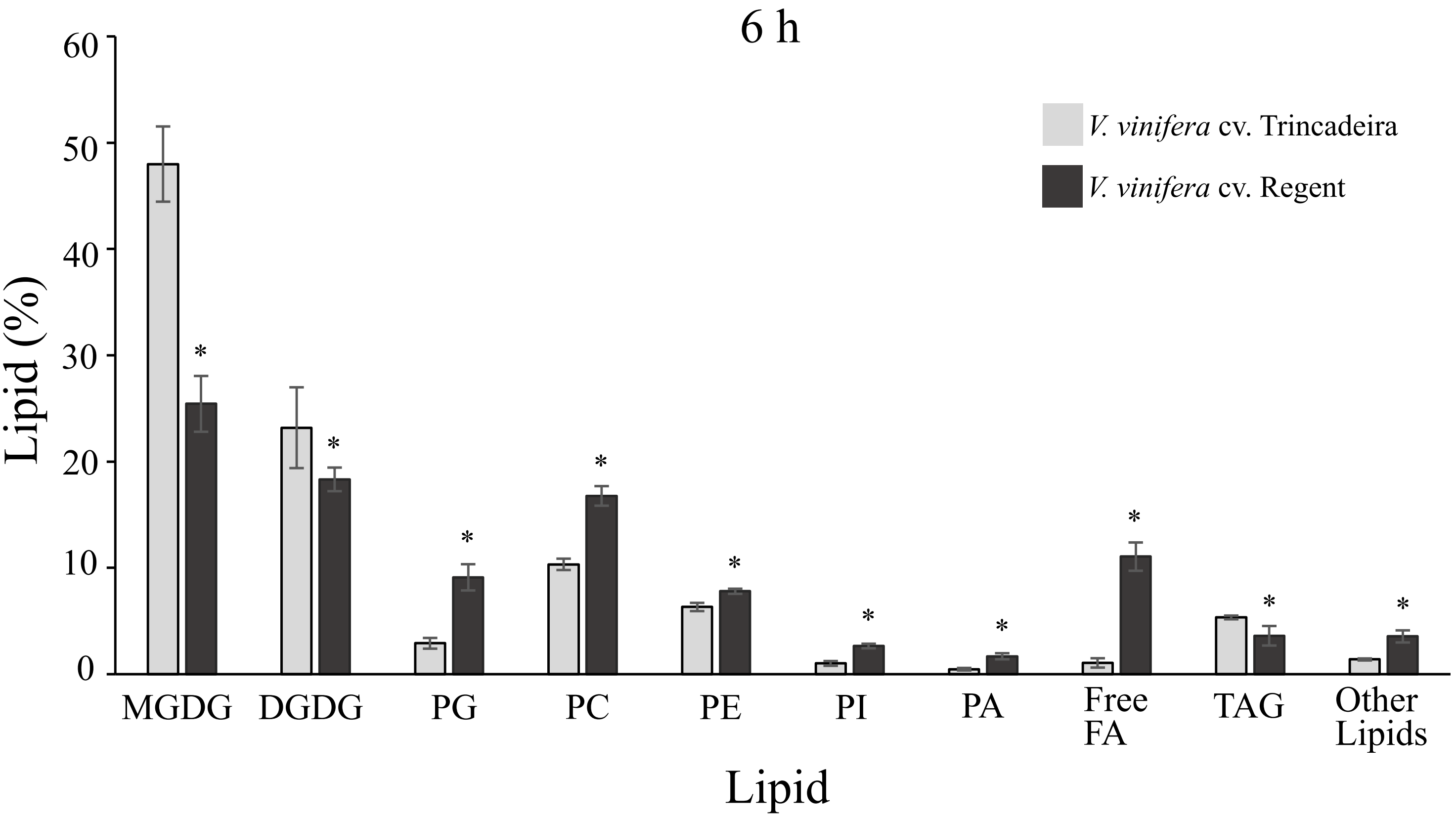

Supplementary Figure S3 - Lipid composition of *V. vinifera* cv. Trincadeira (light grey) and Regent (dark grey) mock inoculated leaves at 6 hours. Values correspond to average relative percentage ± standard error, n = 3; Asterisks indicate significant differences ($p<0.05$). Abbreviations: monogalactosyldiacylglycerol (MGDG), digalactosyldiacylglycerol (DGDG), phosphatidylglycerol (PG), phosphatidylcholine (PC), phosphatidylethanolamine (PE), phosphatidylglycerol (PI), phosphatidic acid (PA), free fatty acids (FA) and triacylglycerol (TAG).

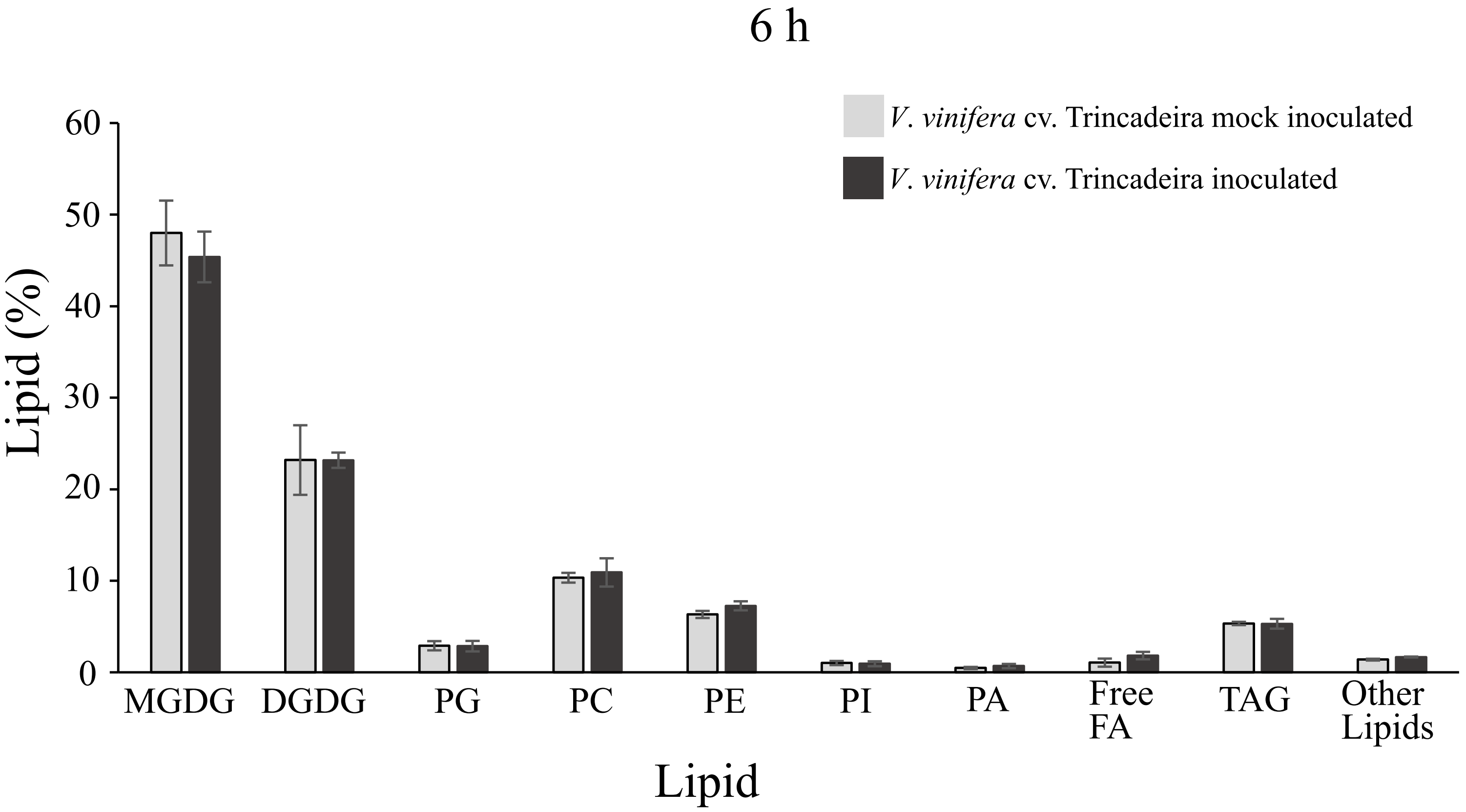

Supplementary Figure S4 - Lipid composition of *V. vinifera* cv. Trincadeira mock inoculated (hm) and inoculated (hpi) leaves at 6 hours. Values correspond to average relative percentage ± standard error, n = 3; Asterisks indicate significant differences (p<0.05). Abbreviations: monogalactosyldiacylglycerol (MGDG), digalactosyldiacylglycerol (DGDG), phosphatidylglycerol (PG), phosphatidylcholine (PC), phosphatidylethanolamine (PE), phosphatidylglycerol (PI), phosphatidic acid (PA), free fatty acids (FA) and triacylglycerol (TAG).

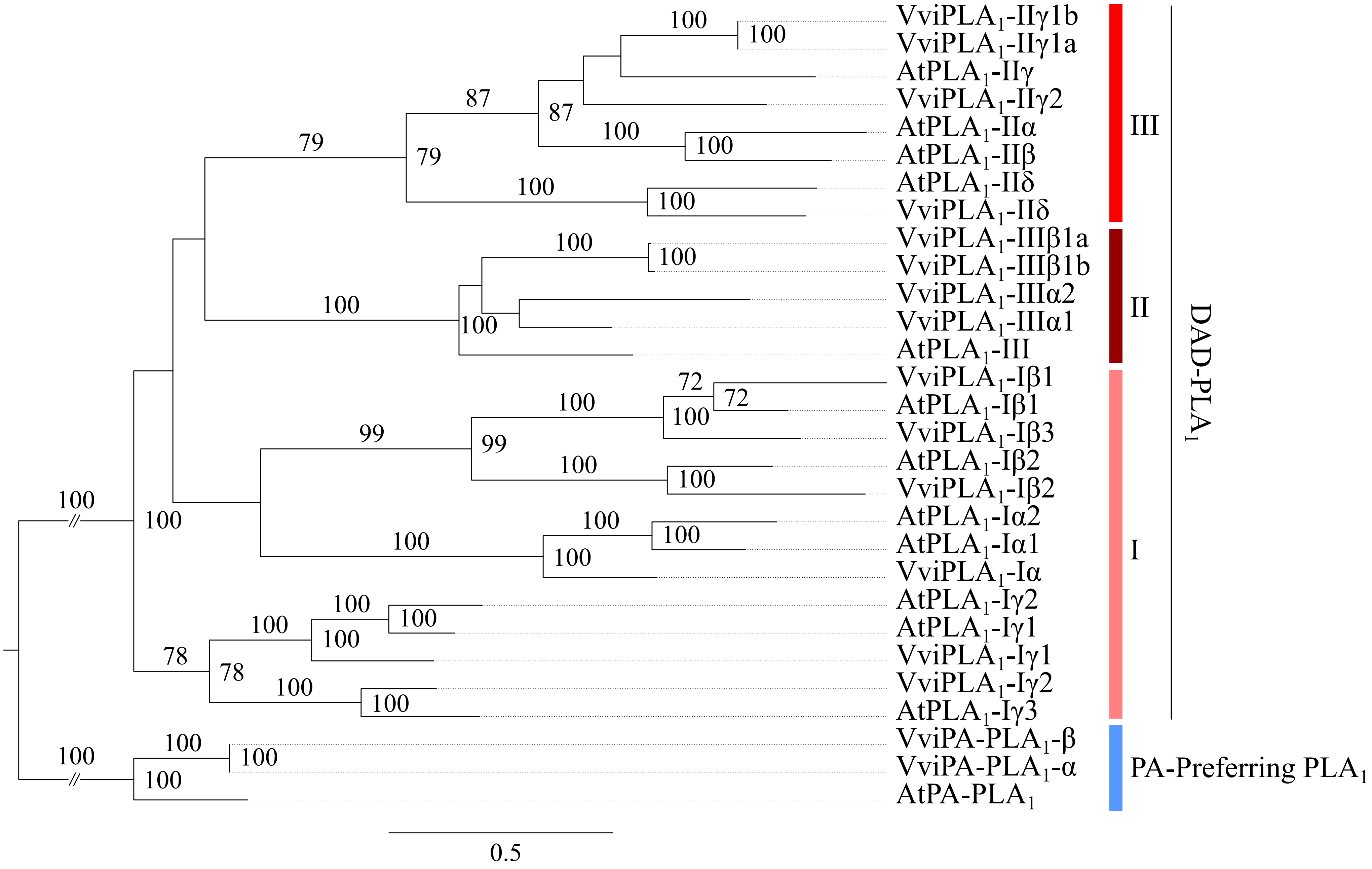

Supplementary Figure S5 - Maximum likelihood phylogenetic tree of the 16 from *Vitis vinifera* PLA_1 proteins and 13 PLA_1 proteins from *Arabidopsis thaliana*. Scale bar represents the number of estimated changes per branch length. The root was truncated with double dash totalling 1.5 changes per branch length.

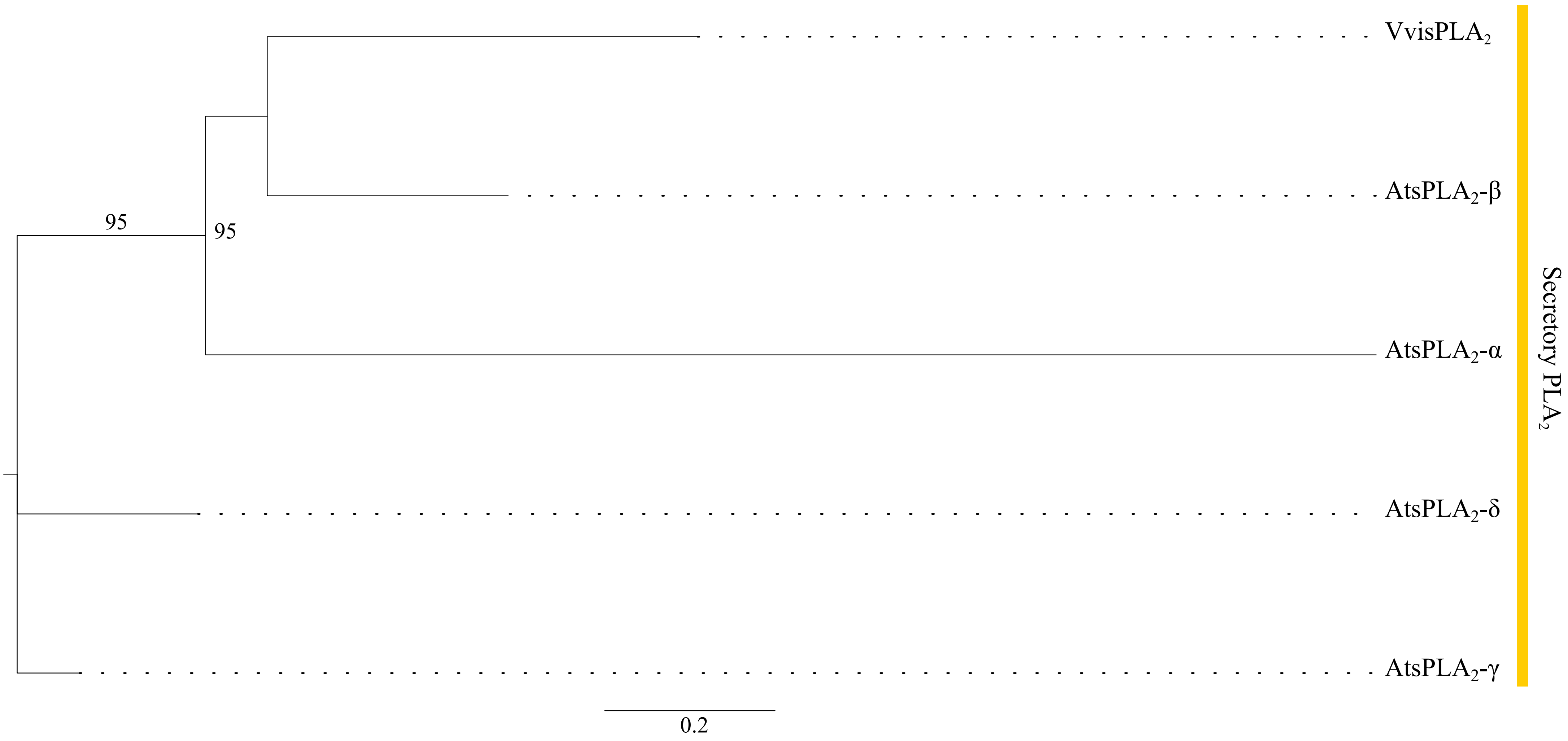

Supplementary Figure S6 - Maximum likelihood phylogenetic tree of the 1 from *Vitis vinifera* secretory PLA proteins and 4 PLA proteins from *Arabidopsis thaliana*. Scale bar represents the number of estimated changes per branch lengt

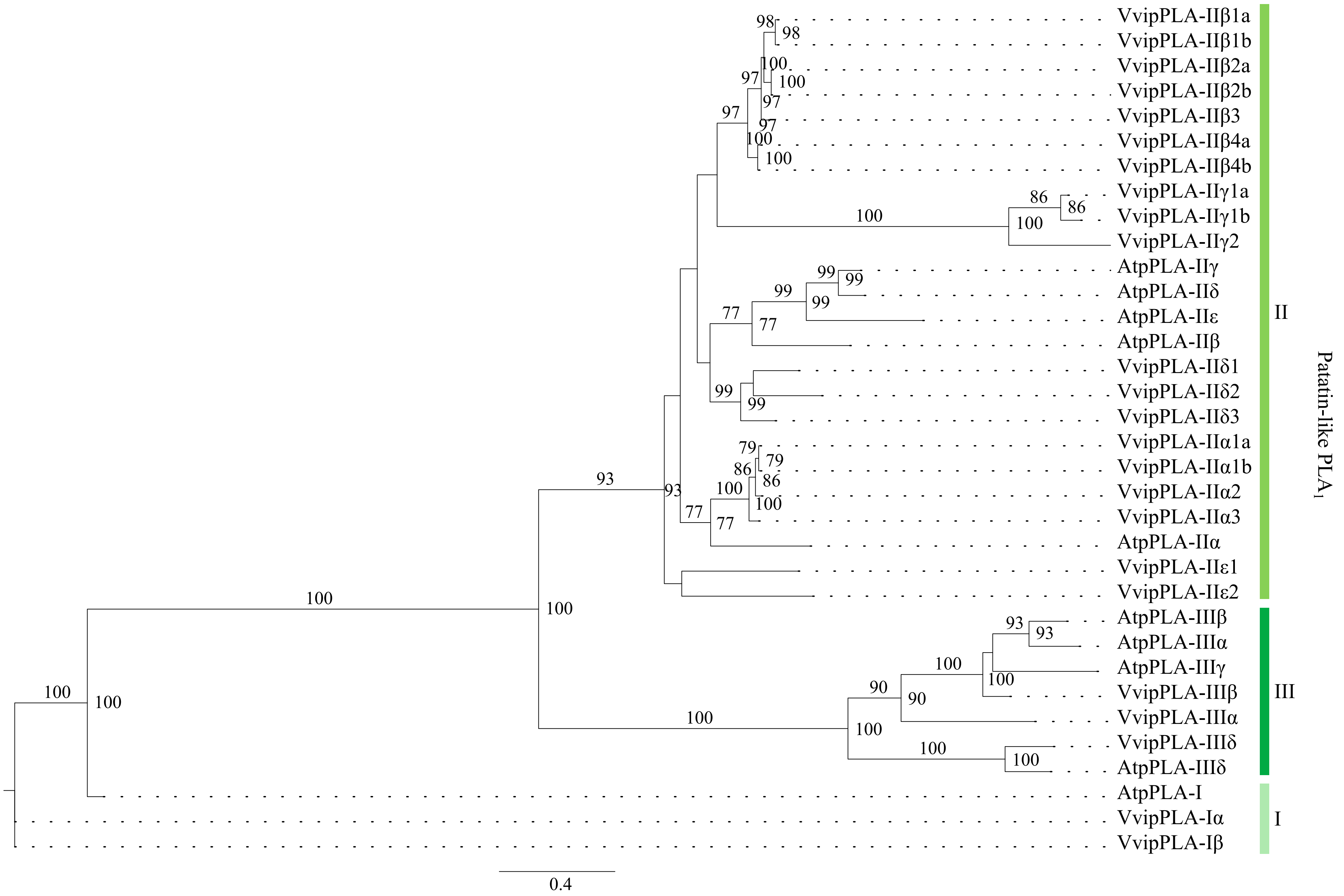

Supplementary Figure S7 - Maximum likelihood phylogenetic tree of the 24 from *Vitis vinifera* patatin-like PLA proteins and 10 PLA proteins from *Arabidopsis thaliana*. Scale bar represents the number of estimated changes per branch length.

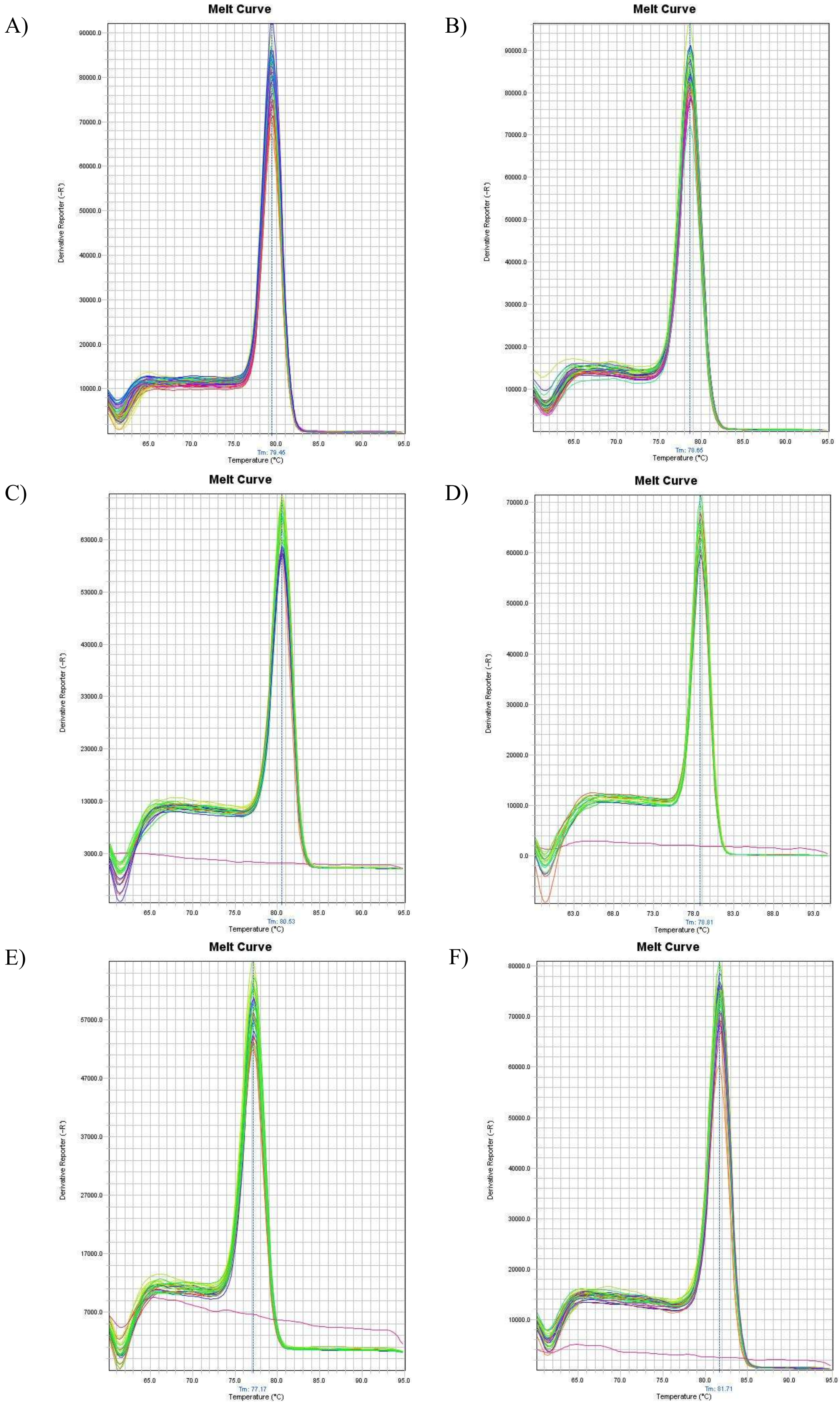
A)
Melt Curve
Derivative Reporter (-R')
Temperature (°C)
Tm: 79.45
B)
Melt Curve
Derivative Reporter (-R')
Temperature (°C)
Tm: 78.65
C)
Melt Curve
Derivative Reporter (-R')
Temperature (°C)
Tm: 80.53
D)
Melt Curve
Derivative Reporter (-R')
Temperature (°C)
Tm: 78.81
E)
Melt Curve
Derivative Reporter (-R')
Temperature (°C)
Tm: 77.17
F)
Melt Curve
Derivative Reporter (-R')
Temperature (°C)
Tm: 81.71

G)

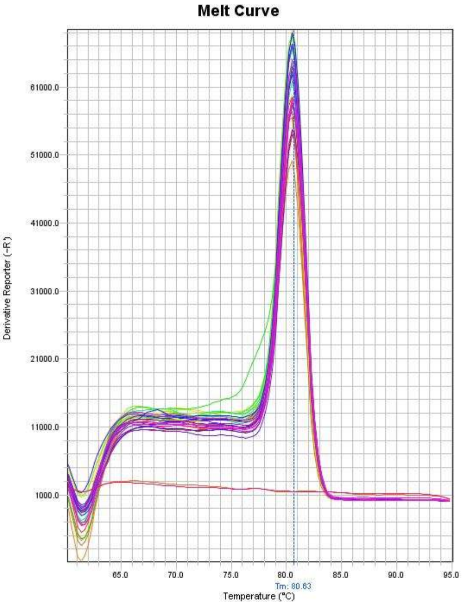

H)

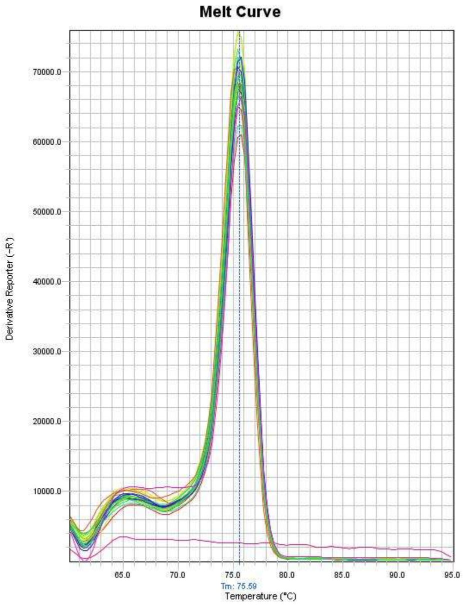

I)

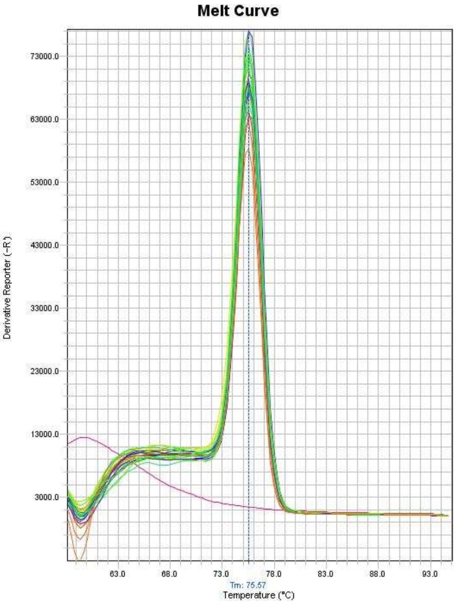

J)

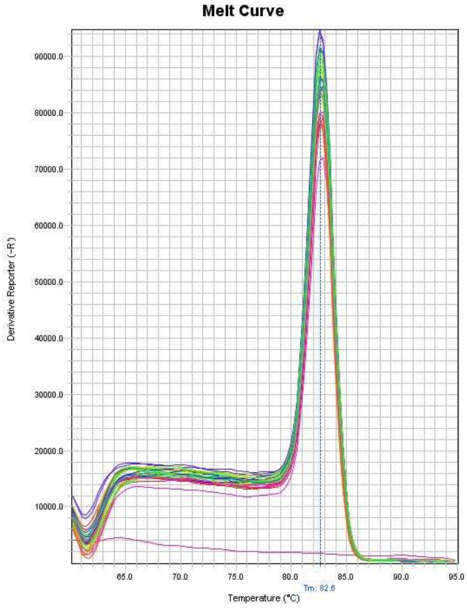

Supplementary Figure S8 - Melting curves of targeted genes. A) *EF1α*; B) *UBQ*; C) *VviPLA1-Iβ1*; D) *VviPLA1-Iγ1*; E) *VviPLA1-IIδ*; F) *VvisPLA2*; G) *VvipPLA-I*; H) *VvipPLA-IIβ*; I) *VvipPLA-IIδ2*; J) *VvipPLA-III*.

Trincadeira		C16:0	C16:1t	C18:0	C18:1	C18:2	C18:3	DBI	C18:3/18:2
**MGDG**	**M**	2.3 ± 0.1	0	0.4 ± 0.1	0.7 ± 0.2	3.6 ± 0.0	93.0 ± 0.2	2.9 ± 0.0	25.8 ± 0.1
	**I**	2.2 ± 0.4	0	0.4 ± 0.0	1.1 ± 0.2	3.7 ± 0.1	92.7 ± 0.5	2.9 ± 0.0	**25.0* ± 0.3**
**DGDG**	**M**	20.4 ± 4.2	0	2.1 ± 0.7	1.1 ± 0.2	3.0 ± 0.4	73.5 ± 5.4	2.3 ± 0.2	25.4 ± 4.9
	**I**	20.5 ± 1.0	0	1.6 ± 0.1	1.0 ± 0.1	2.5 ± 0.1	74.3 ± 0.8	2.3 ± 0.0	29.6 ± 0.7
**PC**	**M**	36.1 ± 1.9	0	5.1 ± 1.0	6.5 ± 0.5	33.5 ± 2.0	18.8 ± 1.4	1.3 ± 0.1	0.6 ± 0.0
	**I**	36.4 ± 4.7	0	5.0 ± 0.8	7.3 ± 0.5	33.6 ± 3.2	17.7 ± 1.8	1.3 ± 0.1	**0.5* ± 0.0**
**PE**	**M**	42.9 ± 6.2	0	3.7 ± 1.3	1.2 ± 1.6	40.7 ± 3.8	12.6 ± 2.2	1.2 ± 0.2	0.3 ± 0.0
	**I**	40.2 ± 3.3	0	2.9 ± 0.0	2.2 ± 0.2	41.8 ± 2.6	12.9 ± 0.9	1.2 ± 0.1	0.3 ± 0.0
**TAG**	**M**	14.4 ± 0.1	0	4.8 ± 0.3	4.4 ± 0.2	42.4 ± 0.1	34.0 ± 0.3	1.9 ± 0.0	0.8 ± 0.0
	**I**	13.7 ± 0.8	0	4.3 ± 0.6	**5.2* ± 0.6**	41.3 ± 0.8	34.3 ± 0.6	1.9 ± 0.0	0.8 ± 0.0
**PG**	**M**	34.7 ± 5.3	34.4 ± 2.4	3.8 ± 0.6	13.8 ± 2.5	4.1 ± 0.8	8.3 ± 2.9	0.4 ± 0.1	2.0 ± 0.3
	**I**	37.6 ± 6.5	30.7 ± 3.5	3.4 ± 1.9	16.8 ± 2.8	4.2 ± 2.5	8.2 ± 2.6	0.4 ± 0.1	1.7 ± 0.4
**Other Lipids**	**M**	32.1 ± 4.2	0	19.8 ± 4.2	12.8 ± 3.3	25.0 ± 4.2	16.5 ± 4.0	1.1 ± 0.2	0.6 ± 0.1
	**I**	40.3 ± 5.0	0	**30.3* ± 3.2**	10.0 ± 1.9	14.5 ± 4.0	7.7 ± 6.3	0.6 ± 0.2	0.7 ± 0.0
**PA**	**M**	28.9 ± 13.3	0	13.9 ± 18.2	0	47.3 ± 25.3	7.9 ± 11.2	1.2 ± 0.7	0.3 ± 0.3
	**I**	24.2 ± 21.8	0	13.7 ± 6.0	4.7 ± 4.9	31.9 ± 16.2	17.1 ± 8.9	1.4 ± 0.6	0.4 ± 0.3
**PI**	**M**	34.4 ± 3.1	0	11.0 ± 7.0	3.8 ± 3.4	19.6 ± 3.1	27.4 ± 2.5	1.3 ± 0.1	1.6 ± 0.3
	**I**	33.6 ± 3.9	0	14.8 ± 6.0	4.3 ± 1.8	18.7 ± 1.9	25.4 ± 4.2	1.1 ± 0.1	1.6 ± 0.3
**Free FA**	**M**	34.7 ± 4.4	0	53.1 ± 5.1	5.8 ± 4.2	4.4 ± 5.0	2.7 ± 5.2	0.2 ± 0.3	0.8 ± 0.1
	**I**	39.6 ± 2.4	0	51.3 ± 2.7	5.5 ± 0.6	5.2 ± 0.7	2.0 ± 3.9	0.2 ± 0.1	0.6 ± 0.7

Supplementary Table S1 - Fatty acids content in each lipid class *V. vinifera* cv. Trincadeira mock inoculated (M) and inoculated (I) leaves with *P. viticola*. Values correspond to average relative percentage ± standard error, n = 3; Asterisks indicate significant differences ($p<0.05$).

Regent		C16:0	C16:1t	C18:0	C18:1	C18:2	C18:3	DBI	C18:3/18:2
MGDG	M	6.5 ± 0.7	0	1.9 ± 0.2	2.2 ± 1.4	3.9 ± 0.1	85.5 ± 2.0	2.7 ± 0.0	22.1 ± 1.0
	I	**3.2* ± 0.1**	0	**0.8* ± 0.0**	**0.6* ± 0.1**	**3.4* ± 0.1**	**92.0* ± 0.2**	**2.8* ± 0.0**	**26.9* ± 0.6**
DGDG	M	24.2 ± 1.1	0	3.4 ± 0.4	1.4 ± 0.1	3.0 ± 0.2	67.9 ± 1.2	2.1 ± 0.1	23.7 ± 2.4
	I	22.0 ± 0.7	0	**1.8* ± 0.1**	**0.8* ± 0.1**	**1.9* ± 0.0**	**73.5* ± 0.6**	**2.2* ± 0.0**	**38.7* ± 0.5**
PC	M	35.5 ± 6.2	0	6.5 ± 1.0	10.7 ± 1.0	28.6 ± 3.4	20.5 ± 4.2	1.3 ± 0.2	0.7 ± 0.1
	I	34.8 ± 0.3	0	**4.2* ± 0.0**	**8.5* ± 0.0**	32.4 ± 0.9	19.9 ± 0.5	1.3 ± 0.0	0.6 ± 0.0
PE	M	41.4 ± 10.0	0	5.9 ± 0.6	4.0 ± 0.6	36.7 ± 7.3	11.8 ± 3.4	1.1 ± 0.2	0.3 ± 0.0
	I	45.5 ± 8.9	0	4.2 ± 0.7	3.7 ± 0.4	37.6 ± 7.6	9.7 ± 2.3	1.1 ± 0.2	0.3 ± 0.0
TAG	M	27.9 ± 0.1	0	16.1 ± 2.5	13.4 ± 2.9	26.8 ± 0.9	15.9 ± 4.4	1.1 ± 0.1	0.6 ± 0.1
	I	**20.4* ± 1.8**	0	**10.1* ± 1.3**	10.1 ± 1.2	**35.5* ± 2.6**	**26.5* ± 3.8**	1.5 ± 0.1	0.8 ± 0.1
PG	M	36.5 ± 2.2	33.9 ± 1.4	4.0 ± 1.6	19.5 ± 1.2	4.4 ± 1.1	4.7 ± 1.9	0.4 ± 0.1	1.1 ± 0.2
	I	35.7 ± 2.6	30.9 ± 1.3	2.1 ± 0.2	19.9 ± 0.6	5.2 ± 1.7	8.1 ± 2.4	0.5 ± 0.1	1.2 ± 0.2
Other Lipids	M	28.4 ± 2.0	0	21.9 ± 1.1	10.3 ± 2.0	23.1 ± 0.0	16.3 ± 1.2	1.1 ± 0.1	0.7 ± 0.1
	I	34.6 ± 6.0	0	**26.4* ± 0.1**	8.1 ± 2.6	**18.2* ± 1.4**	**12.7* ± 2.1**	0.8 ± 0.1	0.7 ± 0.1
PA	M	27.6 ± 4.7	0	16.1 ± 2.6	10.0 ± 2.4	34.7 ± 6.6	11.6 ± 2.9	1.1 ± 0.2	0.3 ± 0.0
	I	32.8 ± 5.0	0	12.1 ± 1.1	6.6 ± 1.8	34.4 ± 4.4	14.9 ± 2.8	1.2 ± 0.1	0.4 ± 0.1
PI	M	52.9 ± 5.0	0	10.9 ± 2.3	4.2 ± 2.1	15.8 ± 3.2	13.5 ± 3.7	0.8 ± 0.2	0.9 ± 0.1
	I	59.2 ± 7.7	0	**8.0* ± 1.2**	3.0 ± 1.8	19.7 ± 3.8	13.1 ± 3.4	0.8 ± 0.2	0.7 ± 0.0
Free FA	M	35.3 ± 0.9	0	56.5 ± 0.0	3.5 ± 0.0	2.8 ± 0.5	2.0 ± 0.5	0.2 ± 0.0	0.7 ± 0.0
	I	**41.0* ± 1.3**	0	**49.8* ± 1.6**	3.4 ± 0.0	2.9 ± 0.3	**3.0* ± 0.1**	0.2 ± 0.0	**1.0* ± 0.1**

Supplementary Table S2 - Fatty acids content in each lipid class of *V. vinifera* cv. Regent mock inoculated (M) and inoculated (I) leaves with *P. viticola*. Values correspond to average relative percentage ± standard error, n = 3; Asterisks indicate significant differences ($p<0.05$).

Family	Group	Proposed nomenclature	Locus	Nucleotide	Protein	Chr.	Introns	Portein length (aa)	Molecular weight (kDa)	pI	Domain (Blast2GO)	Subcellular location
**DAD PLA$_1$**	**I**	VviPLA$_1$-Iα	LOC100242894	XM_002268106.2	XP_002268142.1	5	0	446	49.8	7.04	Lipase_3	Chloroplast
		VviPLA$_1$-Iβ1	LOC100242763	XM_002270956.4	XP_002270992.2	15	0	434	48.7	9.48	Lipase_3	Chloroplast
		VviPLA$_1$-Iβ2	LOC100247003	XM_010648953.2	XP_010647255.1	unknown	0	528	59.1	9.04	Lipase_3	Chloroplast
		VviPLA$_1$-Iβ3	LOC100259863	XM_002271029.4	XP_002271065.3	15	0	389	43.3	8.82	Lipase_3	-
		VviPLA$_1$-Iγ1	LOC100249474	XM_002281871.3	XP_002281907.1	7	1	514	58.2	7.07	Lipase_3	Chloroplast
		VviPLA$_1$-Iγ2	LOC100260417	XM_002285331.3	XP_002285367.1	6	0	502	57.7	8.59	Lipase_3	Chloroplast
	**II**	VviPLA$_1$-IIγ1a	LOC100242927	XM_002266946.3	XP_002266982.2	10	2	369	41.2	6.73	Lipase_3	Mitochondrial
		VviPLA$_1$-IIγ1b	LOC100242927	XM_019222491.1	XP_019078036.1	10	2	406	45.5	6.73	Lipase_3	Mitochondrial
		VviPLA$_1$-IIγ2	LOC100254098	XM_019223761.1	XP_019079306.1	12	3	403	45.7	8.93	Lipase_3	Mitochondrial
		VviPLA$_1$-IIδ	LOC100267811	XM_002281059.4	XP_002281095.2	12	0	470	53.1	5.84	Lipase_3	Chloroplast
	**III**	VviPLA$_1$-IIIα1	LOC100266852	XM_002272744.3	XP_002272780.2	10	0	524	59.7	9.51	Lipase_3	-
		VviPLA$_1$-IIIα2	LOC100242997	XM_002272522.4	XP_002272558.2	10	0	511	58.8	7.32	Lipase_3	-
		VviPLA$_1$-IIIβ1a	LOC100251427	XM_010657396.2	XP_010655698.1	10	0	510	58.0	7.07	Lipase_3	Chloroplast
		VviPLA$_1$-IIIβ1b	LOC100261664	XM_010657395.2	XP_010655697.1	10	0	510	58.1	7.72	Lipase_3	Chloroplast
**PA-preferring PLA$_1$**		VviPA-PLA$_1$-α	LOC100260107	XM_002275576.3	XP_002275612.1	18	20	971	109.4	5.03	DDHD	vacuole membrane / plasmodesma
		VviPA-PLA$_1$-β	LOC100260107	XM_010647575.2	XP_010645877.1	18	20	963	108.5	5.02	DDHD	vacuole membrane / plasmodesma
**Secretory PLA2**		VvisPLA$_2$	LOC100261227	XM_002282786.3	XP_002282822.1	11	3	151	16.3	8.70	-	integral component of membrane
**Patatin-like PLA**	**I**	VvipPLA-Iα	LOC104880454	XM_010657059.2	XP_010655361.1	10	18	1067	118.5	5.88	Arm / Patatin domain	-
		VvipPLA-Iβ	LOC104880454	XM_019222598.1<br>XM_010657058.2	XP_019078143.1<br>XP_010655360.1	10	18	1316	146.2	5.83	Arm / Patatin domain	-
	**II**	VvipPLA-IIα1a	LOC100257463	XM_002282445.3	XP_002282481.1	18	6	413	45.0	5.97	Patatin domain	Cytoplasm / membrane
		VvipPLA-IIα1b	LOC109121419	XM_010666282.2	XP_010664584.1	18	6	413	45.1	5.97	Patatin domain	Cytoplasm / membrane
		VvipPLA-IIα2	LOC100266120	XM_002282561.3	XP_002282597.1	18	6	413	45.0	5.81	Patatin domain	Cytoplasm / membrane
		VvipPLA-IIα3	LOC100241962	XM_002284535.4	XP_002284571.1	18	6	425	46.5	5.28	Patatin domain	Cytoplasm / membrane
		VvipPLA-IIβ1a	LOC100252227	XM_002282330.3	XP_002282366.2	18	6	411	45.4	6.35	Patatin domain	Cytoplasm / membrane
		VvipPLA-IIβ1b	LOC100264244	XM_019216450.1	XP_019071995.1	18	6	411	45.3	6.35	Patatin domain	Cytoplasm / membrane
		VvipPLA-IIβ2a	LOC100248840	XM_002282379.2	XP_002282415.2	18	6	406	44.9	6.98	Patatin domain	Cytoplasm / membrane
		VvipPLA-IIβ2b	LOC100260810	XM_002282396.2	XP_002282432.1	18	7	406	44.9	6.98	Patatin domain	Cytoplasm / membrane
		VvipPLA-IIβ3	LOC100255738	XM_002282404.1	XP_002282440.1	18	6	406	44.9	5.95	Patatin domain	Cytoplasm / membrane
		VvipPLA-IIβ4a	LOC100254055	XM_019216314.1	XP_019071859.1	18	7	406	44.8	8.86	Patatin domain	Cytoplasm / membrane
		VvipPLA-IIβ4b	LOC100267749	XM_019216433.1	XP_019071978.1	18	6	406	44.7	8.85	Patatin domain	Cytoplasm / membrane
		VvipPLA-IIγ1a	LOC100260531	XM_010653988.2	XP_010652290.1	7	12	425	47.0	6.27	Patatin domain	Cytoplasm / membrane
		VvipPLA-IIγ1b	LOC100264086	XM_002272007.3	XP_002272043.1	7	6	426	47.1	6.33	Patatin domain	Cytoplasm / membrane
		VvipPLA-IIγ2	LOC100260531	XM_002271666.3	XP_002271702.1	7	12	424	46.6	9.21	Patatin domain	Cytoplasm / membrane
		VvipPLA-IIδ1	LOC100249295	XM_002281762.4	XP_002281798.1	7	6	407	4.40	5.31	Patatin domain	Cytoplasm / membrane
		VvipPLA-IIδ2	LOC100245674	XM_002277282.3	XP_002277318.1	7	6	402	44.2	5.48	Patatin domain	Cytoplasm / membrane
		VvipPLA-IIδ3	LOC100250815	XM_002277269.2	XP_002277305.1	7	7	407	44.5	5.86	Patatin domain	Cytoplasm / membrane
		VvipPLA-IIε1	LOC100244172	XM_002262906.3	XP_002262942.1	unknown	5	397	43.6	5.96	Patatin domain	Cytoplasm / membrane
		VvipPLA-IIε2	LOC100262840	XM_019221012.1	XP_019076557.1	7	6	398	44.1	7.16	Patatin domain	Cytoplasm / membrane
	**III**	VvipPLA-IIIα	LOC100252820	XM_002276301.3	XP_002276337.2	10	1	484	52.4	9.18	Patatin domain	-
		VvipPLA-IIIβ	LOC100246118	XM_010660108.2<br>XM_019224348.1	XP_010658410.1<br>XP_019079893.1	13	4	458	49.7	6.91	Patatin domain	-
		VvipPLA-IIIδ	LOC100263127	XM_002277845.4	XP_002277881.1	7	1	385	42.0	8.85	Patatin domain	-

Supplementary Table S3 - General features of grapevine PLA superfamily. Proposed grapevine PLA nomenclature, gene locus, protein and nucleotide accessions (from NCBI and CRIBI), chromosome locations, intron number, protein length, molecular weight, isoelectric point (pI), domain and subcellular prediction are represented.

PLA gene	Nucleotide	PLA Location (Mb)	Chr.	*Rpv*	RPV Location (Mb)	Associated marker	References
VviPLA1-Iα	XM_002268106.2	20.6	5	*Rpv14*		GF05-13	Ochssner et al. 2016
VviPLA$_1$-Iα2	XM_002281871.3	0.2	7	*Rpv7*	11.4	UDV-097	Bellin et al. 2009
VviPLA1-Iγ1	XM_002277845.4	1.3					
VvipPLA-IIδ3*	XM_002277269.2	17					
VvipPLA-IIδ2*	XM_002277282.3	17		*Rpv9*	16.6	CCoAOMT	Moreira et al. 2011
VvipPLA-IIδ1*	XM_002281762.4	17					
VvipPLA-IIγ1a	XM_010653988.2	4.3					
VvipPLA-IIγ1b	XM_002272007.3	4.3					
VviPLA1-IIδ	XM_002281059.4	2.6	12	*Rpv1*	10.3	VVIb32	Merdinoglu et al. 2003
				Rpv6	20.4	VMC8G9	Marguerit et al. 2009
VviPLA1-IIγ2	XM_019223761.1	3.1		*Rpv13*	10.0	VMC1G3.2	Moreira et al. 2011
VviPA-PLA1-α	XM_002275576.3	0.9	18	*Rpv2*			Wiedemann-Merdinoglu et al. 2006; Bellin et al. 2009
VviPA-PLA1-β	XM_010647575.2	0.9					
VvipPLA-IIα3	XM_002284535.4	9.1					
VvipPLA-IIα2	XM_002282561.3	9.1					
VvipPLA-IIα1b	XM_010666282.2	9.1		*Rpv3*	24.9<br>26.9	UDV-112<br>UDV-305<br>VMC7f2	Welter et al. 2007; Bellin et al. 2009
*VvipPLA-IIβ4a**	XM_019216314.1	9.1					
VvipPLA-IIα1a	XM_002282445.3	9.2					
VvipPLA-IIβ3	XM_002282404.1	9.2					
VvipPLA-IIβ2b	XM_002282396.2	9.2		*Rpv15*			Pap et al. (in preparation)
VvipPLA-IIβ2a	XM_002282379.2	9.2					
VvipPLA-IIβ1b	XM_019216450.1	9.3					
VvipPLA-IIβ1a	XM_002282330.3	9.3					

Supplementary Table S4 - Grapevine PLA genes and chromosomal location of "Resistance to *Plasmopara viticola* (*Rpv*)" loci. Bold indicate PLA selected for further studies, bold and asterisks indicate the PLA with putative involvement in defence.

Gene name			Abbreviation	NCBI Accession Number	Primer Sequence	Amplicon length (bp)	Ta (°C)	Tm (°C)	Amplification efficiency (E)
**Elongation Factor 1-alpha**			*EF1α*	XM_002284888.2	Fw: GAACTGGGTGCTTGATAGGC<br>Rev: ACCAAAATATCCGGAGTAAAAGA	149	60	79.45	1.89
**Ubiquitin-conjugating enzyme**			*UBQ*	XM_002284161.3	Fw: GAGGGTCGTCAGGATTTGGA<br>Rev: GCCCTGCACTTACCATCTTTAAG	75	60	78.65	1.97
**Phospholipase A1**	**Group I**		*VviPLA1-Iβ1*	XM_002270956.4	Fw: TCCCTAGTGCTCATTCCGAT<br>Rev: TTGAAGAAGACTGGCGACCT	122	58	78.81	2.05
			VviPLA1-Iγ1	XM_002281871.3	Fw: ACTGCGGGACCTGTAGATTT<br>Rev: TTAGGCCATCGGGACTTCTT	142	60	80.53	2.00
	**Group II**		*VviPLA1-IIδ*	XM_002281059.4	Fw: ATGAGGAGGGGAGTAGCAG<br>Rev: TCTGGGGTCACCTGAAGTG	104	60	77.17	1.94
**Secretory Phospholipase A2**			*VvisPLA2*	XM_002282786.3	FW: CAGGTGCGCCAATAGGACA<br>Rev: CGGCGACTGCGAAGAAGAA	89	60	81.71	1.91
**Patatin-like phospholipase A**	**Group I**		*VvipPLA-Iα**	XM_010657059.2	FW: TTGTGCCTGAACCCCGTGT<br>Rev: CCTTTCTTCCCAACCTGTCT	109	60	80.48	2.06
	**Group II**		*VvipPLA-IIα1/2**	XM_002282445.3	FW: TTCATACCTCCATCGCTCC<br>Rev: CCTCTTATTCCACCTCCAT	Not detected due to the low presence of transcript			
			*VvipPLA-IIβ**	XM_019216314.1	FW: CTGCAGCACCAACATACTTT<br>Rev: CTGCGACACCACCATCAATA	96	58	75.59	1.90
			VvipPLA-IIδ1	XM_002281762.4	FW: ATAACAACGGGTCCACTCCA<br>Rev: ATCCGAGGGCCTGAAATACA	Not detected due to the low presence of transcript			
			VvipPLA-IIδ2	XM_002277282.3	FW: GACTCCGTGAGTCAATGGC<br>Rev: TGGGGTGAAGAAGTGGTCAA	116	60	75.57	2.02
			VvipPLA-IIδ3	XM_002277269.2	FW: CGGAATCAAGCCTTTTTACC<br>Rev: TCATATTTTGGCCCTGTTAG	Not detected due to the low presence of transcript			
	**Group III**		*VvipPLA-IIIβ*	XM_010660108.2<br>XM_019224348.1	FW: ACGTTGAAGCCGGTGTTGAT<br>Rev: GGAAGTCGAAGCTGTCTGTT	100	60	82.6	1.92

Supplementary Table S5 - Reference and target genes transcripts primer sequences, amplicon length, amplification efficiency, annealing and melting temperature are represented.
